# Network analysis combined with pharmacological evaluation strategy to reveal the mechanism of Tibetan medicine Wuwei Shexiang pills in treating rheumatoid arthritis

**DOI:** 10.3389/fphar.2022.941013

**Published:** 2022-07-18

**Authors:** Qingxiu He, Xiaoyan Tan, Sang Geng, Qinyun Du, Zhaoqing Pei, Yingrui Zhang, Shaohui Wang, Yi Zhang

**Affiliations:** ^1^ State Key Laboratory of Southwestern Chinese Medicine Resources, School of Ethnic Medicine, Chengdu University of Traditional Chinese Medicine, Chengdu, China; ^2^ State Key Laboratory of Southwestern Chinese Medicine Resources, School of Pharmacy, Chengdu University of Traditional Chinese Medicine, Chengdu, China; ^3^ Affiliated Hospital of University of Tibetan Medicine, University of Tibetan Medicine, Lasa, China; ^4^ State Key Laboratory of Southwestern Chinese Medicine Resources, Innovative Institute of Chinese Medicine and Pharmacy, Chengdu University of Traditional Chinese Medicine, Chengdu, China

**Keywords:** Tibetan medicine, Wuwei Shexiang pills, rheumatoid arthritis, network analysis, GC-MS, UPLC-Q-TOF/MS

## Abstract

Tibetan medicine is an important part of traditional Chinese medicine and a significant representative of ethnic medicine in China. Tibetan medicine is gradually recognized by the world for its unique curative effects. Wuwei Shexiang pills (WPW) has been widely used to treat “Zhenbu” disease (Also known as rheumatoid arthritis) in Tibetan medicine, however, its potential bioactive ingredients and mechanism for RA treatment remain unclear. In this study, we used a combination of gas chromatography-mass spectrometry (GC-MS), ultra-performance liquid chromatography coupled with quadrupole time-of-fight mass spectrometry (UPLC-Q-TOF/MS), network analysis and experimental validation to elucidate the potential pharmacodynamic substances and mechanisms of WPW in the treatment of rheumatoid arthritis (RA). The results showed that songoramine, cheilanthifoline, saussureanine C, acoric acid, arjunolic acid, peraksine, ellagic acid, arjungenin and other 11 components may be the main activities of WPW in the treatment of RA. PIK3CA, AKT, MAPK, IL-6, TNF, MMP1, MMP3, and CDK1 are considered as core targets. PI3K-AKT, MAPK, apoptosis, cell cycle, and other signaling pathways may be the key pathways for WPW to play a role in the treatment of RA. Furthermore, we validated the underlying molecular mechanism of WPW predicted by network analysis and demonstrated its possible mechanism through *in vivo* animal experiments. It was found that WPW could significantly improve the degree of paw swelling, and reduce ankle joint diameter and arthritis index. Further histomorphological analysis showed that WPW could reduce the degree of synovial tissue inflammation and ankle joint cartilage damage. Meanwhile, WPW could down-regulate the levels of IL-6, IL-1β, and IL-17, and increase the levels of IL-10 and IL-4 in the serum of AA rats. TUNEL staining confirmed that WPW could significantly promote the apoptosis of synovial cells. Moreover, the immunohistochemical results showed that WPW decreased the expression of PI3K, AKT, MAPK, MMP1, MMP3, CDK1, and Bcl-2, as well as increased the expression of Bax protein. In conclusion, we successfully combined GC-MS, UPLC-Q-TOF/MS, network analysis, and experimental validation strategies to elucidate the inhibition of inflammation by WPW in AA model rats *via* PI3K/AKT, MAPK, cell cycle and apoptotic pathways process. This not only provides new evidence for the study of potential pharmacodynamic substances and the mechanism of WPW in the treatment of RA, but also provides ideas for the study of other Tibetan medicine compound preparations.

## 1 Introduction

Tibetan medicine originated in the Qinghai-Tibet Plateau of China. It is a complete theoretical system, unique therapeutic methods, and a medical system with strong ethnic characteristics formed by the Tibetan people through long-term practice (Wang et al., 2022b). Tibetan medicine has a long history and there are still a large number of classic works with far-reaching influence, such as “The Four Medical Tantras” and “Jingzhu Materia Medica” (Fu et al., 2020). Rheumatoid arthritis (RA) is a major and difficult disease characterized by progressive joint damage caused by chronic synovitis (Scott et al., 2010). The incidence of RA is high, currently, RA affects about 0.5%–1.0% of the global population, in particular, the risk of developing RA is 2–3 times higher in women than in men (Huang et al., 2021; Jin et al., 2017). Tibetan medicine has unique advantages in treating rheumatism, RA and gout (Wang S. et al., 2021). RA is called “Zhenbu (གྲུམ་བུ།)” in Tibetan medicine. According to Tibetan medicine, “Zhenbu” was caused by “Huangshui” disease, the pathological mechanism of which was the imbalance of the three gastric fires and the passage of the dross into the liver, which further affected the function of “Chiba” (མཁྲིས་པ།) and caused incomplete separated blood to enter the gallbladder, resulting in “Huangshui” (ཆུ་སེར།) all over the body and accumulation in the joints, inducing “Zhenbu” disease (Su et al., 2021).

Wuwei Shexiang pills, also known as Wupeng pills (WPW, Tibetan name: ཁྱུང་ལྔ།), is included in the first part of the 2020 edition of the Chinese Pharmacopoeia and the Classic Tibetan medicine book “The Four Medical Tantras”. The formula is composed of *Terminalia* chebula Retz (Combretaceae; Chebulae fructus, 300 g), *Aucklandia lappa* Decne (Asteraceae; Aucklandiae radix, 100 g), *Moschus berezovskii* Flerov (Moschidae; Moschus, 10 g), *Aconitum pendulum* Busch (Ranunculaceae; Radixaconiti, 300 g), *Acorus calamus* L (Acoraceae; Acori calami rhizome, 60 g). And it has anti-inflammatory, analgesic and wind-dispelling effects (Gongbu et al., 2013). It is clinically used in Tibetan medicine to treat “Zhenbu” disease and has a good effect (Ge and Nima, 2021). However, the potential pharmacodynamic substances and mechanisms of WPW against RA have not been elucidated. The pharmacodynamic substance of the compound is the key to the modernization of TCM/Tibetan medicine, which is the basis of safety, effectiveness and quality control of compound preparation of TCM/Tibetan medicine (Wang X. et al., 2021; Xu et al., 2021). Zhu et al. used HPLC to determine the content of Aconitum alkaloids in Wuwei Shexiang pills, and the results showed that the concentrations of aconitine in the range of 3.28–82.0 μg ml^−1^ (r = 0.9998), hypoaconitine in the range of 3.2–80.0 μg ml^−1^ (r = 0.9998) and mesaconitin in the range of 3.2–80.0 μg ml^−1^ (r = 0.9998) showed a good linear relationship (Zhu et al., 2012). Lu et al. used HPLC to analyze the content of costunolide and dehydrocostuslactone in Wuwei Shexiang pills, and it was found that their concentrations were 0.92 mg/g and 1.67 mg/g respectively (Lu and Feng, 2011). Fan et al. used TLC and HPLC methods to identify and determine the content of muscone in this formula, and the results showed that muscone has a good linear relationship in the range of 0.08–1.28 μg (Fan et al., 2007). All in all, the current studies on the pharmacodynamic substances of this prescription mainly focus on the content determination of a certain component or the whole single component, which cannot fully explain the real pharmacodynamic value of WPW. With the development of modern science and technology, some studies on pharmacodynamic substances of traditional Chinese medicine/ethnic medicine have achieved a lot in practice (Zhang F. X. et al., 2021; Jiao et al., 2021; Shan et al., 2021), especially the emergence of artificial intelligence technologies such as network pharmacology (Gao et al., 2021) and systems biology (Joshi et al., 2021), which provide technical support for revealing the complex pharmacodynamic substances and mechanism of traditional Chinese medicine.

In this study, Wuwei Shexiang pills, a classic prescription of Tibetan medicine, was used as the research object. The strategy combining gas chromatography-mass spectrometry (GC-MS), ultra-performance liquid chromatography coupled with quadrupole time-of-fight mass spectrometry (UPLC-Q-TOF/MS), network analysis, and experimental verification was used to explore the potential pharmacodynamic substances of WPW and clarify their possible mechanism of action, which is conducive to guiding the in-depth development and clinical application of WPW. In addition, it also provides ideas for revealing the effective substances of other Tibetan medicine compounds to treat diseases and realizing the quality control of higher-level compound preparations.

## 2 Materials and methods

### 2.1 Materials and chemicals

Chemical reference standards of cholic acid (DSTDD004201), chebulagic acid (DST200927-064), chebulic acid (DST200610-244), corilagin (DST210311-012), ethyl gallate (DSTDM006301), benzoic acid (DSTDB012001), linoleic acid (DST200611-211), betulin (DSTDB002701), aconitine (HR1001S4), benzoylmesaconine (DSTDB005601), benzoylaconitine (DSTDB005501), benzoylhypaconine (DSTDB005701), hypaconitine (DSTDC005801), mesaconitine (DSTDX002502), methyl caffeate (DST210427-054) and costunolide (DSTDM003001) were acquired from Chengdu Desite Biotechnology Co.,. (Chengdu, China), and the purity of all standards were above 98%. Ellagic acid (111959-201903, purity: HPLC ≥98%) was purchased from China National Institute for Standard Products and Drug Control (Beijing, China). Deoxyaconitine was bought from Chenguang Biotechnology Company (Chengdu, China). Agilent 7890A-5975C Gas chromatography-mass spectrometry and Agilent GC-MSD workstation were purchased from Agilent Technologies (Santa Clara, USA). ACQUITY UPLC I-Class Ultra High-Performance Liquid Chromatography system, Xevo G2-XS QTOF High-resolution time of flight mass spectrometer, MassLynxV 4.2 Data acquisition and analysis workstation were obtained from Waters Corporation (Milford, MA, United States).

### 2.2 Sample extraction and preparation of GC-MS and UPLC-Q-TOF/MS

Wuwei Shexiang pills (Specifications: 0.3 g per 10 pills.) were purchased from Tibetan Xiongbalaqu Shenshui Tibetan Medicine Co., LTD (Tibet, China). For the extraction and preparation of GC-MS samples, the Chinese patent medicine WPW sample (15 g) was finely ground and passed through the second sieve. Further, the pills were put into a 250 ml triangular bottle, and 150 ml of n-hexane was added for 45 min of ultrasound. The solution was filtered out by filter paper, and the remaining medicinal materials were washed with n-hexane. The obtained oil-like extract was dehydrated by adding anhydrous sodium sulfate, centrifuged for 1min at 12,000 r min^−1^, dissolved in 1 ml chromatographic hexane, diluted 60 times, and filtered by a microporous filter membrane (0.22 μm), and the test solution was obtained.

For the extraction and preparation of UPLC-Q-TOF/MS samples, the WPW sample (5 g) was accurately weighed and placed in a 100 ml conical flask, then the ultrasonic extraction was performed twice with 6 times the amount of 70% ethanol, and the concentrated extract was dissolved in acetonitrile and placed in a 5 ml flask, then 1 ml constant volume into 10 ml measuring bottle, and the solution was then filtered through a 0.22 μm microporous membrane. The standard stock solutions were prepared by dissolving appropriate amounts of the reference standards in acetonitrile.

### 2.3 GC-MS analysis

The determination was performed on an Agilent HP-5MS capillary mass spectrometry column with 5% Phenyl Methyl Silox (30 m × 250 μm×0.25 μm). The carrier gas is helium (He), carrier gas velocity: 1 ml min^−1^; The heating procedure is as follows: the initial column temperature is 50°C and kept for 0.5 min; In the first stage, the temperature was increased to 150°C at 5°C min^−1^ for 0 min. In the second stage, the temperature was kept at 6°C min^−1^ L 240°C for 2 min. In the third stage, the temperature increased to 250°C at 7°C min^−1^. Split injection, split ratio of 4:1, Injection volume: 1 μl. The conditions of mass spectrometry were as follows: the ion source was EI, the electron energy was 70 eV, the ion source temperature was 230°C, the four-stage rod temperature was 150°C, the solvent delay was 4 min, and the data were collected in full scan mode, and the mass scan range was *m/z* 12–550.

### 2.4 UPLC-Q-TOF/MS analysis

The chemical constituents of WPW were identified by UPLC-Q-TOF/MS. The chromatographic separation was performed on a Waters Acquity BEH-C18 column (2.1 mm × 100 mm, 1.7 μm). Mobile phase A: 0.1% formic acid aqueous solution, mobile phase B: acetonitrile; Flow rate, 0.3 ml min^−1^; Gradient elution (0–2 min, 2%–6%B; 2–10 min, 6%–15%B; 10–25 min, 15%–28%B; 25–35 min, 28%–40%B; 35–45 min, 40%–50%B; 45–50 min, 50%–60%B; 50–53 min, 60–70%; 53–55 min, 70–74%; 55–60 min, 74–80%; 60–70 min, 80–85%; 70–73 min, 85–95%; 73–74 min, 95–95%; 74–75 min, 95–2%; 75–77 min, 2–2%); Column temperature, 35°C; The detection wavelength, 235 nm; Injection volume, 2 μl. Xevo G2-XS QTOF with Electrospray ionization ion (ESI) source was used for mass spectrometry analysis, and the mass spectrometry data were collected under negative and positive ionization modes. The source parameters were set as follows: Capillary voltage: 3 kV, Taper hole voltage: 40 V, Ion source temperature: 150°C, Desolvation temperature: 450°C, Desolvent flow velocity: 800 L Hr^−1^, Scanning range: *m*/*z* 50–1,200, Scan mode: MSe.

### 2.5 Network analysis

#### 2.5.1 WPW active ingredient screening and potential targets prediction

Using the chemical components of *Terminalia* chebula Retz (Combretaceae; Chebulae fructus), *Aucklandia lappa* Decne (Asteraceae; Aucklandiae radix), *Moschus berezovskii* Flerov (Moschidae; Moschus), *Aconitum pendulum* Busch (Ranunculaceae; Radixaconiti), *Acorus calamus* L (Acoraceae; Acori calami rhizome) retrieved from CNKI (https://www.cnki.net/) and PubMed (https://pubmed.ncbi.nlm.nih.gov/) databases as a supplement to the chemical composition of WPW. Then SwissADME (http://www.swissadme.ch/index.php) and SwissTargetPrediction (http://www.swisstargetprediction.ch/) databases were used to screen the active ingredients and predict the potential targets with the threshold conditions were GI absorption = high, Druglikeness≥2 Yes and Probability≥0.1, respectively. Finally, the drugs-active components-potential targets network of WPW was constructed by Cytoscape software (Version 3.7.1).

#### 2.5.2 Target acquisition of WPW for RA treatment

GeneCards (https://www.genecards.org/) database was performed to obtain targets of RA, and the overlap targets of RA and potential targets of active components of WPW obtained by R software (R package: mainly ggplot2 [version 3.3.3]) are the potential targets of WPW for RA treatment.

#### 2.5.3 PPI interaction network construction and analysis

The potential targets of WPW for RA treatment were imported into the STRING (https://cn.string-db.org/) database for Protein-protein interaction (PPI) network analysis, and the interaction score was medium confidence. Then the results obtained from the STRING database are put into Cytoscape software to make a PPI network map. At the same time, Cytoscape plug-ins Cytohubba and MCODE were used to analyze the PPI interaction network.

#### 2.5.4 GO and KEGG enrichment analysis

R software (R package: UpSetR [version 1.4.0]) was used to obtain the overlapping targets of Cytohubba and MCODE plug-in analysis results, namely the key targets of WPW for RA treatment, and then R software was utilized for GO and KEGG enrichment analysis (R package: org. Hs.eg.db [version 3.10.0], ggplot2 [version 3.3.3], clusterProfiler [version 3.14.3]), with the species source limited to *Homo sapiens*, and filter condition was p. adj<0.05 and q value<0.2. Furthermore, the key “target-pathway” network map was visualized by Cytoscape software.

### 2.6 *In Vivo* pharmacological validation

#### 2.6.1 Animals and ethics statement

SPF healthy male SD rats (weight 180–220 g) were provided by Chengdu EnsiWeier Biotechnology Co., Ltd (Chengdu, China, approval number: SCXK (Xiang)2019–0004). All animals were kept in a controlled environment with a temperature of 23 ± 2°C and humidity of 50 ± 5%. Also, keep the indoor light and dark cycles alternating for 12 h/12 h. All animals get free food and water. All procedures for animal experiments were approved by the Experimental Animal Ethics Committee of Chengdu University of Traditional Chinese Medicine and implemented in accordance with the Guidelines for the Care and Use of Laboratory Animals published by the US National Institutes of Health (revised 1996) (Ethical approval number: 2018-15).

#### 2.6.2 AA rat model preparation and animal administration

36 Male SD rats were fed adaptively for 1 week and then randomly divided into six groups with six rats in each group: normal control group (Control), model control group (Model), MTX (1.05 mg/kg) group and WPW high (WPW-H, 18.9 mg/kg), medium (WPW-M, 9.45 mg/kg) and low (WPW-L, 4.725 mg/kg) dose treatment group. It should be noted that the high, medium and low doses of WPW were obtained by the conversion of the clinical dose of WPW. All drugs were dissolved in distilled water and intra-gastric administration (Gavage is given once a day, and each rat was given 1 ml/100 g at a time). The AA model establishment and treatment dose regimen are shown in Figure 4A. In brief, Rats were injected intradermally with a single dose of 0.1 ml complete Freund’s adjuvant (CFA) into the posterior toe area of the hind paw to establish an adjuvant arthritis rat model, and the control group was given the same amount of normal saline. AA induction day was taken as day 0. After 10 days of modeling, except for the MTX group was given twice a week, the other rats were given drugs for 30 consecutive days. Then the rats were sacrificed on the 40th day, and the visceral organs, blood samples, and ankle joints were collected for subsequent analysis.

#### 2.6.3 Arthritis assessment

Bodyweight, left paw swelling, ankle diameter and arthritis index of rats in each group were measured at the planned time (day 0, 5, 10, 15, 20, 25, 30, 35, and 40), respectively. The arthritis index scoring criteria were as follows: 0, no visible signs of arthritis (swelling or erythema); 1, swollen toes or erythema; 2, red and swollen paws; 3, severe swelling and redness of the ankle; 4, severe swelling of the whole leg and all toes, and no bearing capacity (Bao et al., 2019). A total of 16 points was given by adding up the scores for all four paws.

#### 2.6.4 ELISA assay

After the experiment, abdominal aorta blood was taken and serum samples were centrifuged at 4°C, 3,500 r min^−1^ for 15 min. Then, the levels of TNF-α, IL-6, IL-1β, IL-17, IL-4, and IL-10 in the serum of rats were detected in strict accordance with the instructions of the ELISA kit.

#### 2.6.5 Histological examination

The ankle joint after decalcification was taken out and dehydrated with gradient alcohol in turn. After that, paraffin embedding was carried out. The paraffin slicer was used to slice the embedded tissue wax blocks with a thickness of 3 μm. Then hematoxylin-eosin (H&E), safranin-fixed green, and toluidine Blue O, staining was performed, respectively. The slices are then placed for conventional transparency and sealed with neutral gum. The pathological features of the ankle joint and the morphology of articular cartilage were observed under a microscope.

#### 2.6.6 Immunohistochemistry

Paraffin sections of the ankle joint were dewaxed and washed with distilled water, then placed in citric acid antigen repair buffer for antigen repair. Next, the samples were further placed in a 3% hydrogen peroxide solution and sealed with serum. Subsequently, a primary antibody-containing PI3K, AKT, MAPK, MMP1, MMP3, CDK1, CDK3, BAX, and BCL-2 were added and incubated overnight at 4 °C. On the second day, the primary antibody was washed and the secondary antibody was added and incubated for 50 min at room temperature. Finally, the images were observed under a microscope after staining with DAB and hematoxylin.

#### 2.6.7 TUNEL staining

Apoptosis of synovial cells in the synovial region of the ankle joint was achieved by TUNEL staining according to the instructions of the TUNEL staining kit and the previously reported method (Wang et al., 2022a). And then photographed by Olympus IX-83 inverted fluorescence microscope (Tokyo, Japan).

### 2.7 Data statistics and analysis

All data were statistically analyzed using Graphpad Prism 9 software, and the data were expressed as mean ± standard error of the mean (SEM). After the homogeneity of variance test, a *t*-test was used for comparison between the two groups. Comparison between multiple groups After one-way ANOVA, the LSD test was used for pairwise comparison of the mean between each group. *p* < 0.05 were considered as statistically significant differences. ^#^ *p* < 0.05, ^##^ *p* < 0.01, ^###^ *p* < 0.01 vs. the Control group; *p < 0.05, **p < 0.01, ***p < 0.01 vs. the Model group.

## 3 Results

### 3.1 Identification of chemical constituents of WPW

**FIGURE 1:**
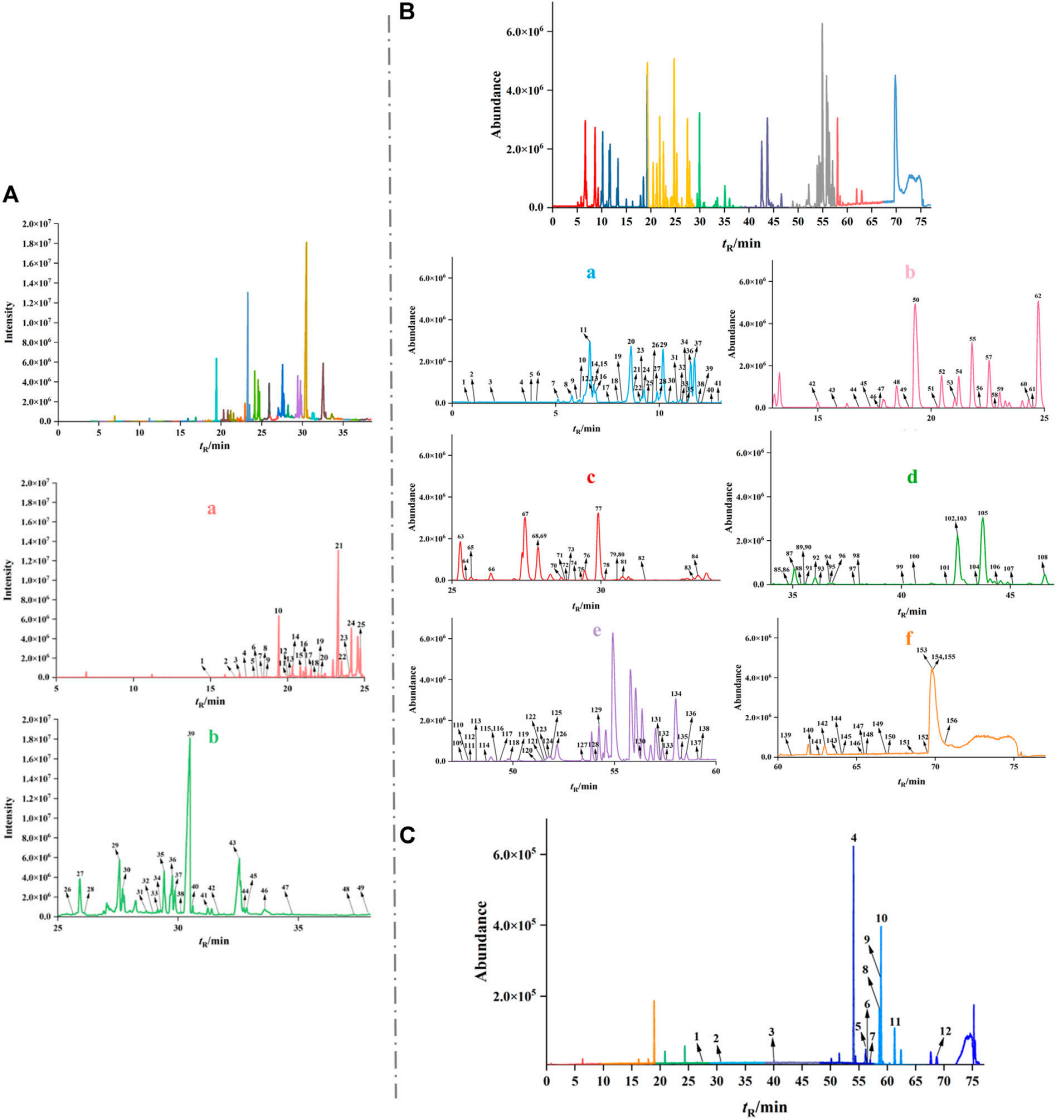
The total ion chromatograms (TICs) of WPW by GS-MS and UPLC-Q-TOF/MS. **(A)** TIC of WPW by GS-MS (a. 5–25 min GC-MS total ion flow diagram b. 25–38 min). **(B)** TIC of WPW in positive ion mode (a. 0–13 min total ion flow diagram, b. 13–25 min, c. 25–34 min, d. 34–47 min, e. 47–60 min, f. 60–77min). **(C)** TIC of WPW in negative ion mode.

GC-MS combined with UPLC-Q-TOF/MS was used to comprehensively analyze and identify the chemical components of WPW. A total of 165 compounds were identified, including 49 sesquiterpenoids, 4 phenylpropanoids, 28 other volatile oils, 67 alkaloids, 10 triterpenoids, and 7 other compounds. The data obtained by GC-MS were searched and matched with the database of the National Institute of Standards and Technology (NIST2014), and a total of 49 volatile oil components were identified. The relative contents of dehydrocostus lactone, β-asarone, and muscone were found to be relatively high by using the peak area normalization method (Figure 1A, Supplementary Table S1). A total of 67 alkaloids, 3 triterpenoids, 51 volatile components, and 7 other compounds were identified in positive ion mode. And a total of 12 chemical constituents including triterpenoids and volatile oils were identified under anion mode by using UPLC-Q-TOF/MS, literature review, and chemical composition database of WPW (Figures 1B–C, Supplementary Tables S1–S5).

### 3.2 Potential target screening and PPI interaction network construction and analysis of WPW and RA

**FIGURE 2:**
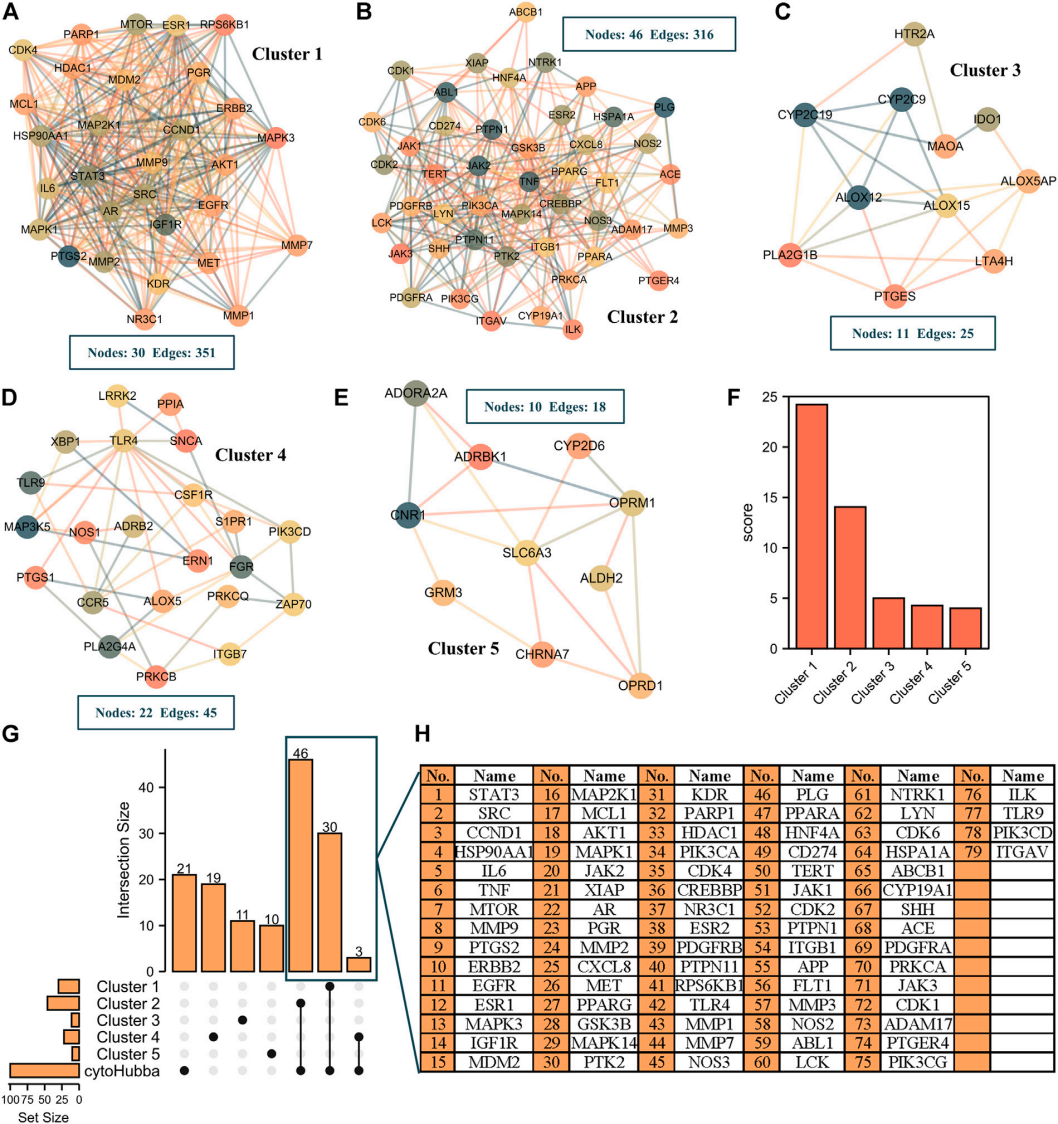
Screening of key targets in the treatment of RA with WPW. **(A–E)** The cluster module with a Score≥4 in the PPI network was obtained by MCODE plug-in Cytoscape. **(F)** The score for the five cluster modules. **(G)** The upset plot was screened by MCODE and Cytohubba. **(H)** A list of 79 key targets in the upset plot.

To identify the potential active components and targets of resveratrol in RA treatment, we further carried out a literature search through CNKI and Pubmed databases and combined with GC-MS and UPLC-Q-TOF/MS technology to obtain the chemical components of WPW. After deleting the repeated chemical components, a library of WPW chemical constituents was constructed. Then 83 active components were obtained by SwissADME screening. Potential targets of 83 active ingredients were further predicted by the SwissTargetPrediction database. Compounds with no target and those with target Probability <0.1 were removed. Finally, 50 active ingredients were included in the subsequent analysis. Among them, there are 6 chemical components from *Acorus calamus* L, and 13 chemical components from *Aucklandia lappa* Decne, 10 chemical components from *Terminalia chebula* Retz, 11 chemical components from *Moschus berezovskii* Flerov, 10 chemical components from *Aconitum pendulum* Busch, and their corresponding targets are 111, 245, 528, 176, 261 respectively. A total of 523 potential targets of WPW were obtained by combining and removing repeated targets, Finally, a drug-active component-potential target network of WPW was constructed using Cytoscape3.7.1 (Supplementary Figure S1A). Further analysis of the network found that there were 11 with Degree ≥50. Including songoramine, cheilanthifoline, quinidine, saussureanine C, linolic acid, acoric acid, songorine, arjunolic acid, peraksine, ellagic acid, and arjungenin. A total of 5017 potential targets of RA were obtained through GeneCards database screening, and further intersection with potential targets of WPW was conducted to obtain 307 intersection targets of WPW and RA, namely potential targets of WPW for RA treatment (Supplementary Figure S1B). The 307 intersection targets were imported into the STRING database to obtain PPI network information, and then the PPI network was visualized by Cytoscape software. The results showed that the PPI network consisted of 307 nodes and 4086 edges, with an average degree value of 26.6 and PPI enriched *p*-value<1.0e-16 (Supplementary Figure S1C). Then, the Maximal Clique Centrality (MCC) algorithm of Cytoscape plug-in Cytohubba was used as the screening method to screen TOP100 nodes from the PPI network (Supplementary Figure S1D). Considering that there may be some differences among different screening conditions, we further screened through the MCODE plug-in and found that there were five Cluster modules with Score≥4 in the PPI network (Figures 2A–E), and their scores were 24.207, 14.044, 5, 4.286, and 4 respectively (Figure 2F). Then, the results obtained by cytohubba and MCODE screening were intersected to obtain 79 targets, namely the key targets of WPW for RA treatment (Figures 2G,H).

### 3.3 GO and KEGG pathway enrichment analyses

**FIGURE 3:**
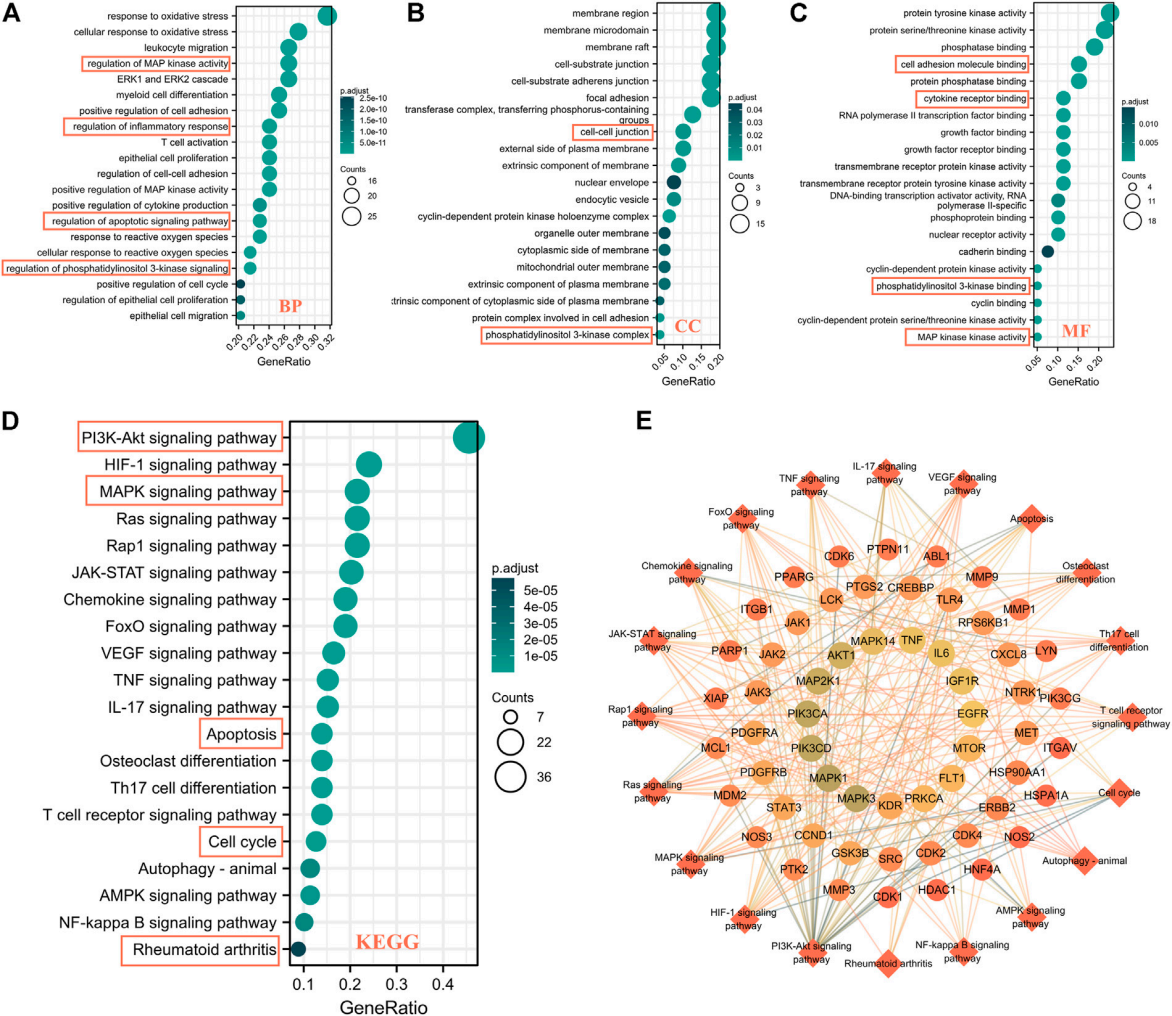
GO and KEGG pathway enrichment analysis of 79 potential key targets of WPW for RA treatment. **(A)** BP. **(B)** CC. **(C)** MF. **(D)** KEGG enrichment pathway. **(E)** A network diagram of target-signaling pathways.

In order to clarify the potential pathway of WPW in RA treatment, we then conducted an enrichment analysis of GO and KEGG pathways for 79 key targets. And the results showed that 2048 biological processes (BP), 61 cell components (CC), 111 molecular functions (MF), and 148 KEGG enrichment pathways met the condition that p. adj<0.05 and qvalue<0.2. BP analysis showed that these 79 key targets were mainly enriched in regulation of MAP kinase activity, regulation of inflammatory response, regulation of apoptotic signaling pathways, regulation of phosphatidylinositol 3-kinase signaling, positive regulation of cell cycle, etc. (Figure 3A). CC analysis showed that it was mainly enriched in membrane rafts, membrane microdomain, cell-cell junction, and phosphatidylinositol 3-kinase complex (Figure 3B). MF analysis results showed that it was mainly enriched in cell adhesion molecule binding, cytokine receptor binding, MAP kinase activity, cyclin binding, phosphatidylinositol 3-kinase binding, etc. (Figure 3C). KEGG pathway enrichment results showed that it was mainly concentrated in the PI3K-AKT signaling pathway, MAPK signaling pathway, apoptosis, cell cycle, rheumatoid arthritis, and other signaling pathways (Figure 3D). Further, 20 KEGG pathways and their mapping targets were constructed. Through the analysis of this network, it was found that PIK3CA, AKT1, MAPK1, IL-6, TNF, MMP1, MMP3, CDK1, and other targets were closely related to these 20 pathways, and this suggested that these targets might be the potential core targets for WPW to play a role in the treatment of RA (Figure 3E).

### 3.4 Effects of WPW on the arthritis index, paw swelling, ankle diameter, body weight, and histopathological changes in AA rats

**FIGURE 4:**
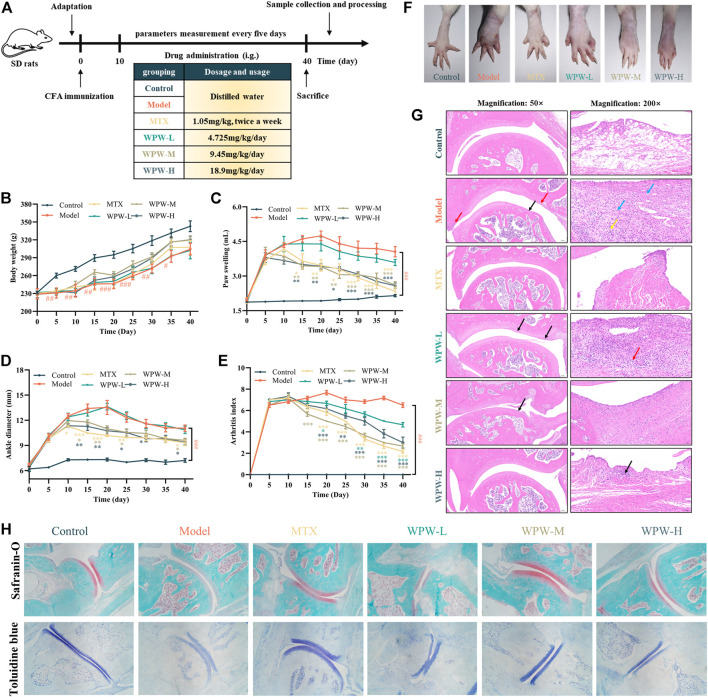
Effect of WPW on the pathological process of RA in AA model rats. **(A)** Experimental schedule of WPW on AA model rats. The arthritis index **(B)**, paw swelling **(C)**, ankle diameter **(D)**, and body weight **(E)** in AA rats (n = 6). **(F)** Representative images of the hind limbs of the different treatment groups at the end of the experiment. **(G)** Histopathological evaluation of the ankle joints of rats in different treatment groups was conducted by H&E staining (n = 3; Magnification: ×50 and ×200; Scale bar: 200 and 50 μm). **(H)** Safranin-O and toluidine blue staining (n = 3; Magnification: ×40).

To investigate the therapeutic effect of WPW on RA, we established AA model rats and administered them. In addition, the body weight, paw swelling, arthritis index, and ankle joint diameter of rats in different treatment groups were also detected (Figure 4A). We found that compared with the model group, the bodyweight of the rats treated with MTX and WPW was significantly improved (Figure 4B). By observing the paw swelling degree of the rats, we found that compared with the control group, the AA model group had red, swollen, ulcerated feet, stiff and deformed joints, and could not bend normally, which indicated that the modeling was relatively successful. Compared with the model group, after 10 days of MTX administration, the degree of foot swelling showed a trend of relief, and at the late stage of administration, the degree of foot swelling was significantly improved without redness, ulceration, and other symptoms. After treatment with different doses of WPW, the degree of redness and swelling of the feet of rats in each group was also significantly improved and showed a significant downward trend (Figures 4C–F). Interestingly, the ankle diameter and arthritis index showed similar trends in all treatment groups (Figures 4D,E). To further observe the pathological changes in ankle joints of AA model rats, hematoxylin-eosin staining (HE) was used for the ankle joints of rats. Compared with the control group, the surface of articular cartilage in the AA model group was slightly less smooth (black arrow), and the edge of articular cartilage was attached by a hyperplastic synovial membrane (red arrow), which showed a large number of fibrocytes and capillary hyperplasia (blue arrow), accompanied by a large number of lymphocyte infiltration (yellow arrow). Compared with the model group, dense connective tissue was observed in the subsynovial layer of rats in the MTX group, but no obvious inflammatory response was observed. There were some differences in sections of different WPW dose groups: in the WPW-M group, some synovial hyperplasia could be observed invading the edge of articular cartilage (black arrow), and dense connective tissue could be seen in the subsynovial layer without an obvious inflammatory response. In the WPW-H group, synovial structure tended to be normal and only a few lymphocytes were observed (black arrow). In the WPW-L group, there is a local proliferation of synovial lining cells (black arrows) and a more diffuse infiltration of lymphocytes (red arrows). In conclusion, after treatment with WPW, all treatment groups showed different degrees of improvement compared with the model group (Figure 4G). In order to further demonstrate that WPW can control inflammation and reduce cartilage destruction, the changes of cartilage in the ankle joints of rats were detected by safranin-O staining and toluidine blue staining. The results showed that, compared with the model group, the degree of cartilage damage in AA rats was effectively reversed after drug administration. Especially in WPW-M and WPW-H groups, the positive staining area of safranin-O and toluidine blue staining were larger and closer to the normal control group (Figure 4H). These results fully indicated that WPW could effectively alleviate synovial inflammation and reduce cartilage destruction in AA model rats.

### 3.5 Effect of WPW on inflammatory factors in the serum of AA model rats

**FIGURE 5:**
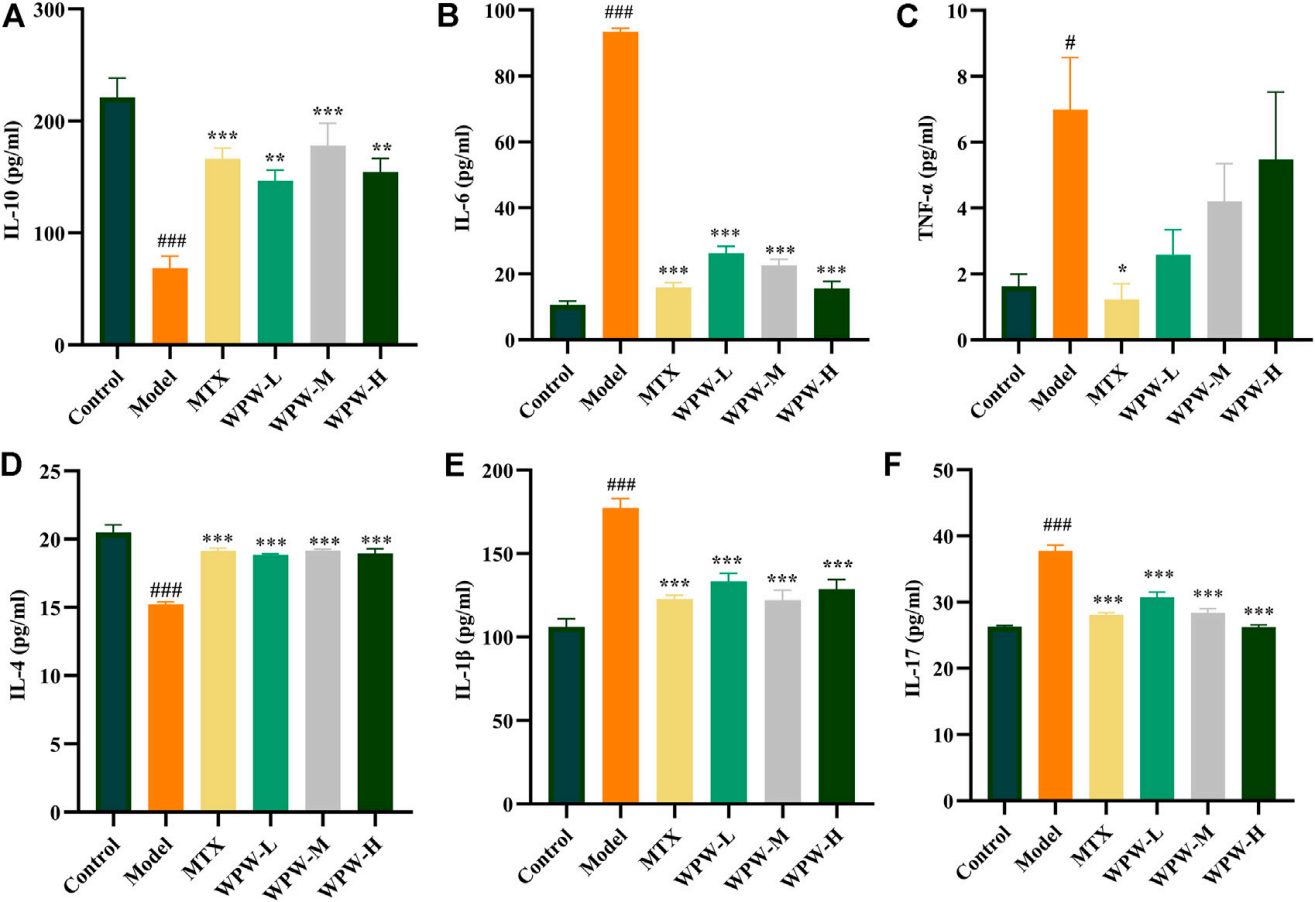
Effect of WPW on inflammatory factors **(A)** IL-10 **(B)** IL-6 **(C)** TNF-α **(D)** IL-4 **(E)** IL-1β **(F)** IL-17 in serum of AA model rats (n = 6).

To clarify the anti-RA effect of WPW, we further detected the levels of pro-inflammatory cytokines IL-6, IL-17, TNF-α, IL-1β, and anti-inflammatory cytokines IL-10 and IL-4 in serum of rats in each group by ELISA. The results showed that compared with the control group, the levels of pro-inflammatory factors (TNF-α, IL-6, IL-1β, IL-17) in the serum of rats after modeling were significantly increased, and the levels of anti-inflammatory factors (IL-10, IL-4) were significantly decreased. After treatment with WPW, the levels of IL-6, IL-17, and IL-1β in the serum of WPW treatment groups were significantly lower than those of rats after modeling, the levels of IL-10 and IL-4 were significantly increased, while TNF-α level decreased, but there was no significant difference (Figures 5A–F). In a word, these results suggested that WPW could exert its anti-RA effect by reducing the secretion of inflammatory cytokines.

### 3.6 Effects of WPW on the PI3K-AKT, MAPK, apoptosis, cell cycle, and other pathway-related proteins

**FIGURE 6:**
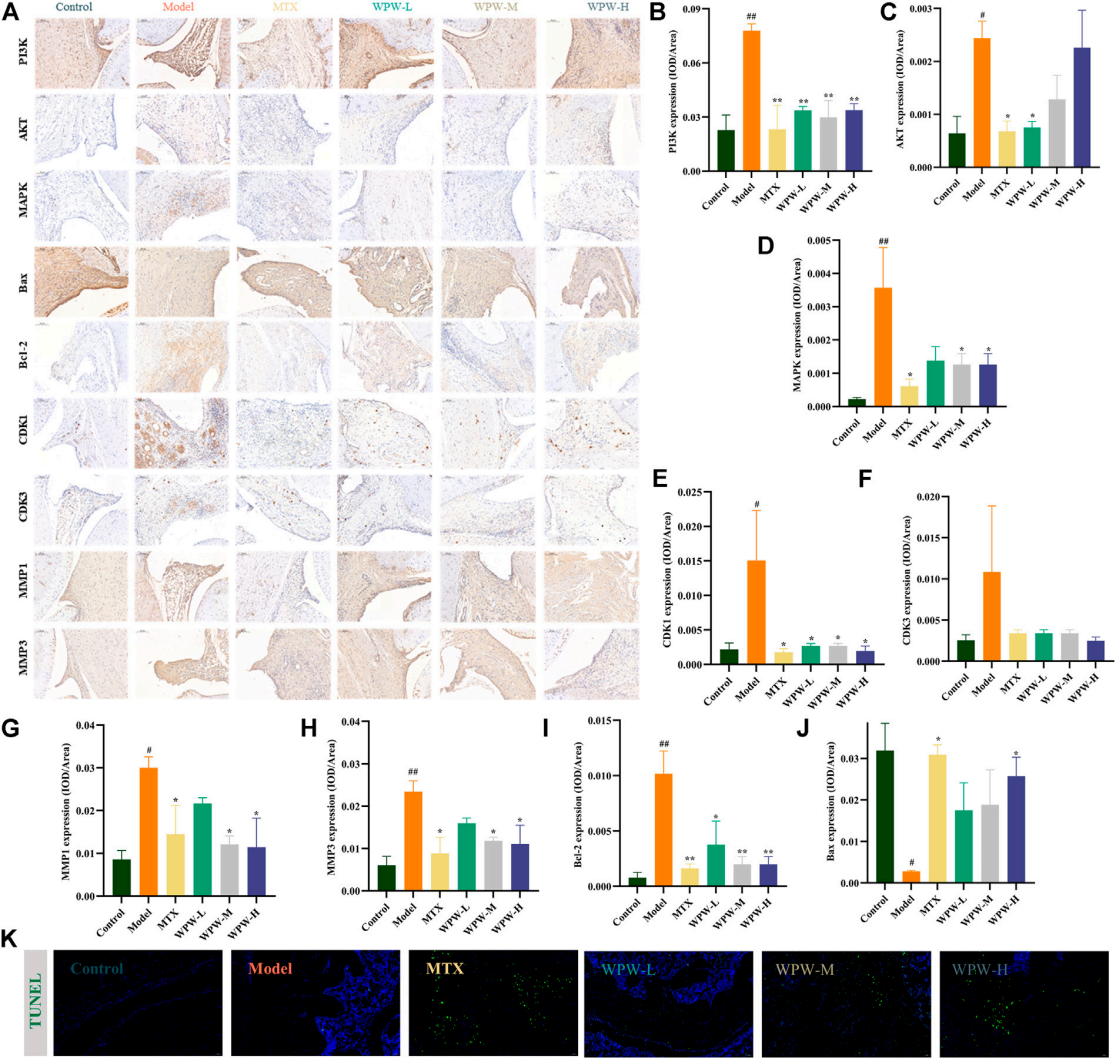
Effect of WPW on protein expression in the synovial region of the ankle joint in AA rats. **(A)** The expression levels of PI3K, AKT, MAPK, Bax, Bcl-2, CDK1, CDK3, MMP1, and MMP3 in arthritic joints were detected by the immunohistochemical method (n = 3; Magnification: ×200; Scale bar: 100 μm). **(B–J)** Quantitative analysis of PI3K, AKT, MAPK, Bax, Bcl-2, CDK1, CDK3, MMP1, and MMP3. **(K)** TUNEL fluorescence staining of ankle joints of rats in different groups (Magnification: ×200; Scale bar: 50 μm).

The results of network analysis suggested that PI3K-AKT, MAPK, apoptosis, cell cycle, and other signaling pathways might be the potential pathway of WPW in the treatment of RA. Therefore, we further detected the expression of PI3K, AKT, MAPK, MMP1, MMP3, CDK1, CDK3, and other core target proteins in the synovial region of the ankle joint by immunohistochemical method (Figure 6A). The results showed that compared with the control group, the positive areas of PI3K, AKT, MAPK, MMP1, MMP3, CDK1, and Bcl-2 increased in the model group. Compared with the model group, the positive expression of MTX and WPW group decreased and tended to a normal level (*p* < 0.05) (Figures 6B–I). Interestingly, Bax positive expression was elevated in the MTX and WPW group compared with the model group (Figure 6J). Thus, we hypothesized that WPW could induce the apoptosis of synovial cells in the ankle joint. As a result, the TUNEL assay was used to detect cell apoptosis in the synovial region of the ankle joint. We found that the fluorescence signal in the synovial region of the ankle joint of AA rats was significantly enhanced after treatment with WPW, indicating that the morphological changes of apoptosis were obvious (Figure 6K). In conclusion, these results further validate the screening results of network analysis and suggest that the therapeutic effect of WPW on RA may be realized by regulating PI3K/AKT, MAPK, cell cycle, and apoptosis signaling pathways.

## 4 Discussion

The pathological manifestations of RA mainly include synovial lining cell proliferation, interstitial inflammatory cell infiltration, microvascular regeneration, pannus formation, cartilage and bone tissue destruction, etc. (Derksen et al., 2017; Rana et al., 2018). Although the exact etiology of RA is still unclear, it is certain that the onset of RA may be related to genetics, infection, sex hormones, and so on (van der Woude and van der Helm-van Mil, 2018; Wang S. et al., 2021). If RA patients cannot be treated in time, progressive joint stiffness, destruction, deformity, and disability may occur in patients, and even complications such as infection, gastrointestinal bleeding, heart, lung, or renal changes may occur in the later stage, which seriously threaten the life and health of patients (Tiniakou et al., 2018; Izuka et al., 2021). Traditional Chinese medicine and ethnic medicine are the precious wealth of the Chinese nation and have unique advantages in the prevention and treatment of some difficult diseases such as RA (Wang Y. et al., 2021; Li et al., 2022). Network pharmacology aims to understand the network biological basis of complex diseases, syndromes, and drug therapy, and has made great progress in predicting targets, understanding the biological basis of diseases and syndromes, network regulation mechanism of compound preparations, and identification of disease biomarkers based on biological network (Ou et al., 2020; Zhang C. et al., 2021; Wang X. et al., 2021; Hou et al., 2021).

As a classic prescription of Tibetan medicine, the efficacy of WPW against RA urgently needs to be revealed. We first identified the volatile components in WPW by GC-MS, and the results showed that the volatile components in WPW mainly included terpenoids, phenylpropanoids, fatty acids, macrolides, and other compounds. Among them, the compounds with high content were dehydrocostus lactone, β-asarone, muscone, and so on. UPLC-Q-TOF/MS technology has been widely used in the separation and analysis of complex systems due to its high sensitivity and analysis efficiency, which has greatly promoted the modernization of Traditional Chinese medicine/ethnic medicine (Duan et al., 2019; Zhou et al., 2021). Therefore, we further analyzed 140 chemical components from WPW using this technique, among which 67 alkaloids were the most. Then, we further supplemented the chemical components of each drug in WPW and screened potential active ingredients and targets through a literature review. Finally, we obtained 50 potential active ingredients and 523 potential targets in the whole WPW prescription. Further analysis found that there were 11 chemical components with a degree value ≥ 50, including songoramine, cheilanthifoline, quinidine, saussureanine C, linolic acid, acoric acid, songorine, arjunolic acid, peraksine, ellagic acid, and arjungenin. Four components were also identified by GC-MS and UPLC-Q-TOF/MS, which were linoleic acid, songorine, arjunolic acid, and arjungenin. Some scholars confirmed that linoleic acid can be used to prevent RA by a Mendelian randomized study (Zhao and Schooling, 2019; Lee, 2020). In addition, other studies have demonstrated that dietary conjugated linoleic acid (CLA) may be an effective means of preventing and alleviating RA, which includes a variety of benefits, including inhibiting endosteal bone absorption, enhancing calcium absorption, affecting inflammatory cytokines, increasing cortical bone formation, and regulating the role and expression of cycloxygenase (COX) (Butz et al., 2007; Hur and Park, 2007). Songorine is a typical active C20-diterpenoid alkaloid derived from the lateral roots of *Aconitum carmichaelii* Debeaux. (Li et al., 2021). The studies of Nesterova et al. and Zhang et al. confirmed that it had good anti-inflammatory and anti-rheumatic activities (Nesterova et al., 2014; Zhang L. et al., 2021). Arjunolic acid is a saponin component with antioxidant, antibacterial, and anti-inflammatory activities (Hemalatha et al., 2010; Ghosh and Sil, 2013). Arjunolic acid is also an effective antioxidant that plays an important role in protecting cells and tissues from the harmful effects of reactive oxygen species (Hemalatha et al., 2010). Arjungenin has certain free radical scavenging activity and also shows a stronger inhibitory effect on hypochlorous acid produced by human neutrophils (Pawar and Bhutani, 2005). In conclusion, we have sufficient evidence to prove that these components may be the important components of WPW to exert an anti-RA effect.

Cytohubba is a plug-in for Cytoscape software to identify hub nodes, while the MCODE plug-in can be used to discover the dense region which interacts in the PPI network (Chin et al., 2014; Liu et al., 2019). So, we further analyzed the 307 targets using these two plug-ins in Cytoscape software, and finally obtained 79 important targets for WPW treatment of RA. Through enrichment analysis of GO and KEGG pathways, it was found that these 79 targets were mainly enriched in PI3K/AKT, MAPK, apoptosis, and cell cycle signaling pathways.

To further verify these predictions, we constructed an AA model to study the therapeutic effect of WPW on RA. As a chronic autoimmune disease, the inflammation of RA cannot subside spontaneously. Therefore, inflammatory homeostasis, which inhibits the development of inflammation and promotes its remission, maybe a new strategy for the treatment of RA (Chen et al., 2019). We found that WPW can exert its therapeutic effect on RA by inhibiting the expression of pro-inflammatory cytokines IL-6, IL-17, IL-1β and promoting the expression of anti-inflammatory cytokines IL-4 and IL-10. This bidirectional regulation may be an advantage of WPW for RA treatment. Recent studies have shown that the PI3K/AKT signaling pathway, as one of the main intracellular signal transduction pathways, widely exists in the synovial membrane and plays an important role in the growth, proliferation, survival, apoptosis, adhesion and migration of fibroblast-like synoviocytes (FLSs) (Li and Wang, 2020; Wang et al., 2022a). Mitogen-activated protein kinase (MAPK) is a serine/threonine-protein kinase family, which has been confirmed to be involved in the RA process by many studies (Liu et al., 2018; Yang et al., 2018; Du et al., 2019; Behl et al., 2021). In addition, in recent years, many molecules that regulate apoptosis and the cell cycle have been suggested to play a role in RA (Perlman et al., 2001; Taranto and Leech, 2006; Collison, 2017). We verified the prediction of network pharmacology by *in vivo* animal experiments and found that WPW decreased the expression of PI3K, AKT, and MAPK in the synovial region of the ankle joint. Moreover, it also decreased the expression of cell cycle-related protein CDK1 and apoptosis-related protein Bcl-2, and increased the expression of Bax. MMP-1 is produced primarily by synovial cells lining joints (Burrage et al., 2006). Other MMPs, such as MMP-3, are also elevated in arthritis, and these enzymes degrade non-collagenous matrix components of joints (Burrage et al., 2006; Di Spigna et al., 2021). We found that WPW significantly inhibited the expression of MMP-1 and MMP-3. These results suggest that WPW can regulate PI3K/Akt, MAPK, apoptosis, and cell cycle pathways to exert its anti-RA effects under inflammatory conditions.

Although at present our preliminary research has been clear about the potential of WPW active ingredients, but these components are based on the analysis of the network, In the future we can further analyze WPW changes in the body, in addition, these ingredients are to work alone or collaborative work, it needs to be further validated through experiments *in vivo* and *in vitro*. It is worth noting that in the network predicted pathway, we found that the anti-RA effect WPW may also be related to osteoclast differentiation, which also provides direction for us to further reveal the mechanism.

## 5 Conclusion

All in all, we used GC-MS, UPLC-Q-TOF/MS technology, network analysis, and animal experimental validation to reveal the potential substance basis and mechanism of Action of Tibetan drug WPW in RA treatment. We found that linoleic acid, songorine, arjunolic acid, arjungenin and other components might be the main chemical components of WPW against RA. PI3K/AKT, MAPK, apoptosis and cell cycle signaling pathways may be the main anti-RA pathways of WPW. The above fully proved that WPW achieves its anti-RA effect through multiple components, multiple targets and multiple signaling pathways, which provides a solid scientific basis for the clinical application of WPW.

## Data Availability

The original contributions presented in the study are included in the article/Supplementary Material, further inquiries can be directed to the corresponding authors.
