# Supplementary material for: Network analysis combined with pharmacological evaluation strategy to reveal the mechanism of Tibetan medicine Wuwei Shexiang pills in treating rheumatoid arthritis
Source: Front Pharmacol. 2022 Jul 18;13:941013. doi: 10.3389/fphar.2022.941013 (PMC9340267; doi:10.3389/fphar.2022.941013)
Supplement: Supplementary file 1 [file DataSheet1.docx]

**Supplementary materials**

**Network analysis combined with pharmacological evaluation strategy to reveal the mechanism of Tibetan medicine Wuwei Shexiang pills in treating rheumatoid arthritis**

Qingxiu He^a,1^, Xiaoyan Tan^b,1^, Sang Geng^c,1^, Qinyun Du^b^, Zhaoqing Pei^d^, Yingrui Zhang^a^, Shaohui Wang^a*^, Yi Zhang^a*^

*^a^ State Key Laboratory of Southwestern Chinese Medicine Resources, School of Ethnic Medicine, Chengdu University of Traditional Chinese Medicine, Chengdu 611137, China*

*^b^ State Key Laboratory of Southwestern Chinese Medicine Resources, School of Pharmacy, Chengdu University of Traditional Chinese Medicine, Chengdu 611137, China*

*^c^ University of Tibetan Medicine, Lasa 850000, China*

*^d^State Key Laboratory of Southwestern Chinese Medicine Resources, Innovative Institute of Chinese Medicine and Pharmacy, Chengdu University of Traditional Chinese Medicine, Chengdu 611137, China*

*** Corresponding authors at:** *State Key Laboratory of Southwestern Chinese Medicine Resources, School of Ethnic Medicine, Chengdu University of Traditional Chinese Medicine, Chengdu 611137, China*. E-mail addresses: zhangyi@cdutcm.edu.cn (Y. zhang), winter9091@163.com (S. Wang).

^1^ These authors contributed equally to this work.

**Figure S1** Potential active ingredients and targets of WPW for RA treatment. **(A)** The network diagram of the drugs-active components-potential targets of WPW. **(B)** Venn diagrams of potential targets of WPW and RA. **(C)** A PPI network of 307 potential common targets. **(D)** Top100 nodes were obtained from the PPI network by the MCC algorithm of the Cytohubba plug-in.


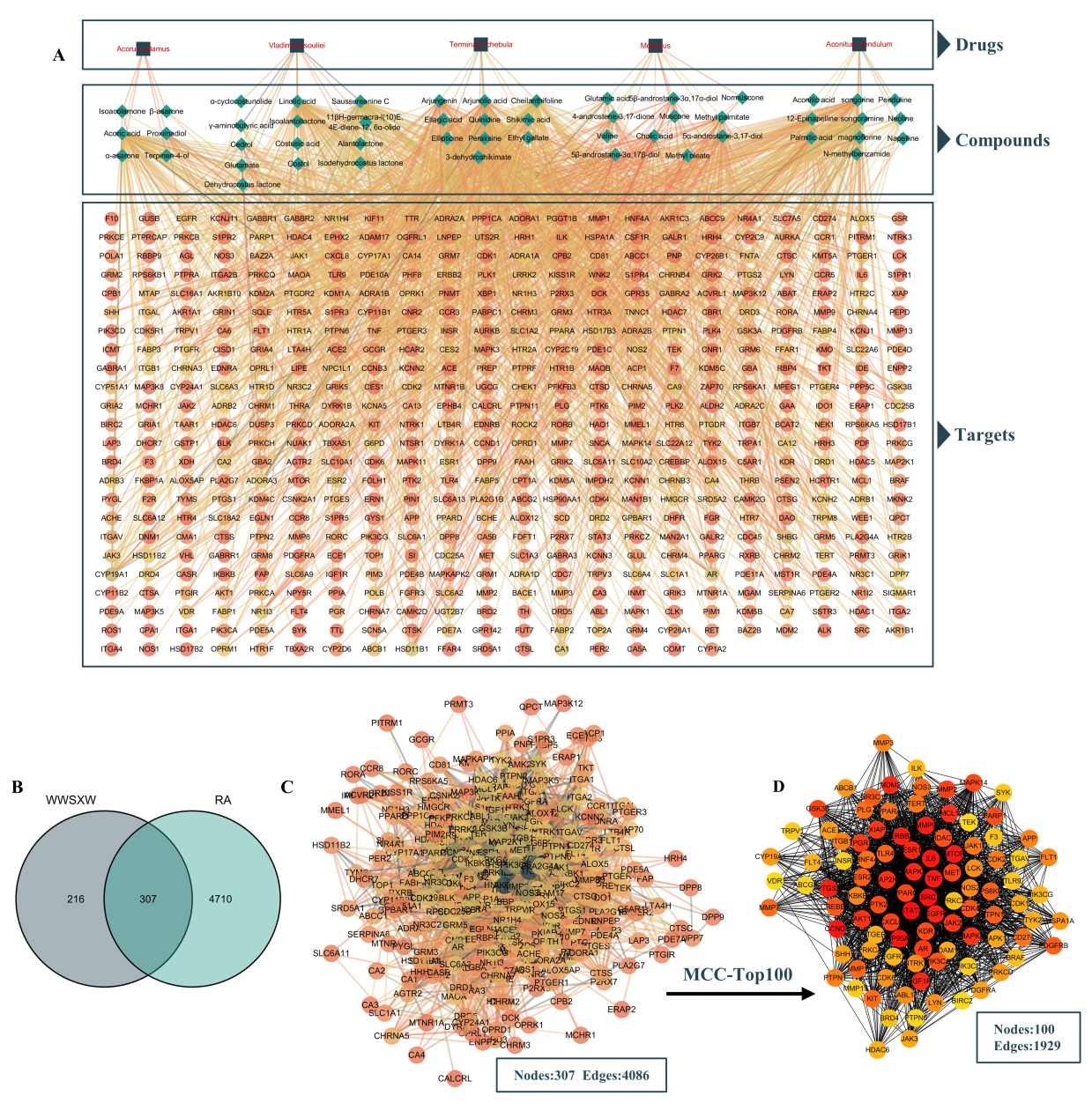


**Tab.S1. Component identification results of WSP under GC-MS**

| **No.** | **Identity** | **Molecular formula** | **T_R_/min** | **Relative content %** |
| --- | --- | --- | --- | --- |
| **1** | Anethole | C_10_H_12_O | 14.95 | 0.02 |
| **2** | (+)-Δ-Cdinene | C_15_H_24_ | 16.61 | 0.01 |
| **3** | Eugenol | C_10_H_12_O_2_ | 16.89 | 0.24 |
| **4** | Copaene | C_15_H_24_ | 17.32 | 0.01 |
| **5** | Tetradecane | C_14_H_30_ | 17.86 | 0.01 |
| **6** | Methyleugenol | C_11_H_14_O_2_ | 18.07 | 0.04 |
| **7** | α-chamigrene | C_15_H_24_ | 18.36 | 0.03 |
| **8** | Caryophyllene | C_15_H_24_ | 18.47 | 0.08 |
| **9** | α-Ionone | C_13_H_20_O | 18.65 | 0.04 |
| **10** | (E)-Methyl isoeugenol | C_11_H_14_O_2_ | 19.44 | 3.24 |
| **11** | Epizonarene | C_15_H_24_ | 19.91 | 0.04 |
| **12** | α-Curcumene | C_15_H_22_ | 20.05 | 0.07 |
| **13** | Aromandendrene | C_15_H_24_ | 20.18 | 0.15 |
| **14** | Epishyobunon | C_15_H_24_O | 20.32 | 0.8 |
| **15** | Shyobunone | C_15_H_24_O | 20.84 | 0.55 |
| **16** | (-)-isoshyobunone | C_15_H_24_O | 21.19 | 0.59 |
| **17** | α-Calacorene | C_15_H_20_ | 21.53 | 0.4 |
| **18** | Elemicin | C_12_H_16_O_3_ | 21.78 | 0.12 |
| **19** | β-calacorene | C_15_H_20_ | 22.00 | 0.2 |
| **20** | γ-Asarone | C_12_H_16_O_3_ | 22.21 | 0.13 |
| **21** | β-Asarone | C_12_H_16_O_3_ | 23.29 | 7.79 |
| **22** | Dehydroxy-isocalamendiol | C_15_H_24_O | 23.51 | 1.04 |
| **23** | heptadeca-1,8,11-triene | C_17_H_30_ | 24.04 | 0.37 |
| **24** | 1,3-Cyclooctadiene | C_8_H_12_ | 24.15 | 2.61 |
| **25** | 1-Isopropyl-4,8-dimethylspirodec-8-en-7-on | C_15_H_24_O | 24.73 | 1.46 |
| **26** | (E)-Ligustilide | C_12_H_14_O_2_ | 25.64 | 0.21 |
| **27** | Isocalamenediol | C_15_H_26_O_2_ | 25.92 | 3.25 |
| **28** | Tetradecanoicacid | C_14_H_28_O_2_ | 26.10 | 0.35 |
| **29** | dehydrosaussurea lactone | C_15_H_20_O_2_ | 27.57 | 5.41 |
| **30** | Muscone | C_16_H_30_O | 27.70 | 2.91 |
| **31** | Eudesma-5,11(13)-dien-8,12-olide | C_15_H_20_O_2_ | 28.69 | 0.69 |
| **32** | Methylester | C_17_H_34_O_2_ | 28.95 | 0.27 |
| **33** | Cembrene | C_20_H_32_ | 29.16 | 0.45 |
| **34** | β-Cyclodihydrocostunolide | C_15_H_22_O_2_ | 29.28 | 0.49 |
| **35** | Dihydrodehydrocostuslactone | C_15_H_20_O_2_ | 29.42 | 3.7 |
| **36** | N-Hexadecanoicacid | C_16_H_32_O_2_ | 29.76 | 3.72 |
| **37** | β-Cyclocostunolide | C_15_H_20_O_2_ | 29.87 | 2.19 |
| **38** | Ethyl palmitate | C_18_H_36_O_2_ | 30.12 | 0.48 |
| **39** | Dehydrocostus lactone | C_15_H_18_O_2_ | 30.48 | 22.84 |
| **40** | Costunolide | C_15_H_20_O_2_ | 30.61 | 0.89 |
| **41** | Thunbergol | C_20_H_34_O | 31.24 | 0.61 |
| **42** | Methyllinoleate | C_19_H_34_O_2_ | 31.70 | 0.15 |
| **43** | Linoelaidicacid | C_18_H_32_O_2_ | 32.55 | 8.83 |
| **44** | Linoleicacidethylester | C_20_H_36_O_2_ | 32.74 | 0.47 |
| **45** | Octadecanoicacid | C_18_H_36_O_2_ | 32.86 | 0.87 |
| **46** | Ent-copalol | C_20_H_34_O | 33.61 | 1.61 |
| **47** | Tetracosane | C_24_H_50_ | 34.72 | 0.24 |
| **48** | Eicosane | C_20_H_42_ | 37.32 | 0.11 |
| **49** | Octadecane | C_18_H_38_ | 37.95 | 0.17 |

**Tab.S2. Mass spectrometry information of reference substance under UPLC-Q-TOF-MS positive ion mode**

| **No.** | **T_R_/min** | **Identity** | **Molecular formula** | **Theoretical (Da)** | **Calculated mass (Da)** | **Error (ppm)** | **Adducts** | **MS Fragmentation** |
| --- | --- | --- | --- | --- | --- | --- | --- | --- |
| 1 | 6.32 | Benzoic acid | C_7_H_6_O_2_ | 122.0367 | 123.0446 | 4.4 | +H | 105.0132 |
| 2 | 10.29 | Ethyl gallate | C_9_H_10_O_5_ | 198.0528 | 199.0599 | -1.2 | +H | 153.0186,125.0234,171.0285 |
| 3 | 15.91 | Methyl caffeate | C_10_H_10_O_4_ | 194.0579 | 195.0654 | 1.3 | +H | 89.0399,163.0397,117.0354,145.0304 |
| 4 | 16.55 | Benzoylmesaconine | C_31_H_43_NO_10_ | 589.2887 | 590.2951 | -1.5 | +H | 540.2565,105.0351,558.2729,508.2304,572.2843,526.2417 |
| 5 | 18.13 | Benzoylaconitine | C_32_H_45_NO_10_ | 603.3043 | 604.3108 | -1.3 | +H | 105.0351,554.2732,572.2843,522.2460,586.2989,540.2565 |
| 6 | 19.59 | Benzoylhypaconine | C_31_H_43_NO_9_ | 573.2937 | 574.3003 | -1.3 | +H | 542.2783,105.0351,510.2471 |
| 7 | 23.86 | Mesaconitine | C_33_H_45_NO_11_ | 631.2992 | 632.3059 | -1.1 | +H | 572.2843,354.1705,512.2678,540.2565,554.2732 |
| 8 | 25.82 | Hypaconitine | C_33_H_45_NO_10_ | 615.3043 | 616.3103 | -2.2 | +H | 556.2879,524.2661,338.1727 |
| 9 | 26.96 | Aconitine | C_34_H_47_NO_11_ | 645.3149 | 646.3204 | -2.8 | +H | 616.3119,586.2989,556.2879,368.1848 |
| 10 | 29.46 | Deoxyaconitine | C_34_H_47_NO_10_ | 629.3200 | 630.327 | -0.4 | +H | 570.3080,538.2792,352.1935 |
| 11 | 44.52 | Costunolide | C_15_H_20_O_2_ | 232.1463 | 233.1532 | -1.6 | +H | 187.1460 |
| 12 | 56.73 | Betulin | C_30_H_50_O_2_ | 442.3810 | 443.3886 | 0.6 | +H | 407.3662 |

**Tab.S3. Mass spectrometry information of reference substance under UPLC-Q-TOF-MS negative ion mode**

| **No.** | **T_R_/min** | **Identity** | **Molecular formula** | **Theoretical (Da)** | **Calculated mass (Da)** | **Error (ppm)** | **Adducts** | **MS Fragmentation** |
| --- | --- | --- | --- | --- | --- | --- | --- | --- |
| 1 | 1.38 | Chebulic acid | C_14_H_12_O_11_ | 356.03796 | 355.0306 | -0.2 | -H | 337.0224,205.0475,193.0139,249.0407,161.0601 |
| 2 | 9.05 | Corilagin | C_27_H_22_O_18_ | 634.08061 | 633.0733 | -0.1 | -H | 301.0009,275.0212 |
| 3 | 10.29 | Ethyl gallate | C_9_H_10_O_5_ | 198.05282 | 197.0453 | -1.5 | -H | 124.0175 |
| 4 | 12.01 | Chebulagic acid | C_41_H_30_O_27_ | 954.09745 | 953.0909 | 0.8 | -H | 301.0009,169.0120,275.0212,125.0229,337.0224 |
| 5 | 12.77 | Ellagic acid | C_14_H_6_O_8_ | 302.00627 | 300.9992 | 0.8 | -H | 283.9935,229.0116 |
| 6 | 35.42 | Cholic acid | C_24_H_40_O_5_ | 408.28757 | 407.2797 | -1.5 | -H | 345.2774,327.2522 |
| 7 | 58.94 | Linoleic acid | C_18_H_32_O_2_ | 280.24023 | 279.2338 | 2.9 | -H | 261.2322 |

**Tab.S4. Component identification results of WPW under UPLC-Q-TOF-MS positive ion mode**

| **Peak no.** | **T_R_/min** | **Identity** | **Molecular formula** | **Theoretical (Da)** | **Calculated mass (Da)** | **Error (ppm)** | **Adducts** | **MS Fragmentation** |
| --- | --- | --- | --- | --- | --- | --- | --- | --- |
| 2 | 2.05 | Salsolinol | C_10_H_13_NO_2_ | 179.095 | 180.102 | 2.1 | +H | 134.8924,152.9054,118.9232,170.9135,84.9610 |
| 3 | 3.84 | Columbianine | C_22_H_35_NO_5_ | 393.252 | 394.26 | 1.7 | +H | 86.0966,184.0735,104.1069,89.0598,133.0883,124.9998,162.9498,95.0887,163.0172,81.0727,119.0883,69.0714,71.0889,84.9610,101.9499,109.1015,118.9232,136.9310,177.1140,79.0579,98.9876,117.0925,156.8901,166.0635,146.9818 |
| 4 | 4.09 | Senbusine A | C_23_H_37_NO_6_ | 423.262 | 424.27 | 1.1 | +H | 184.0735,86.0966,104.1069,124.9998,146.9818,162.9498,156.8901,264.2725,321.2127,295.2019,370.2197 |
| 5 | 5.11 | Senbusine B | C_23_H_37_NO_6_ | 423.262 | 424.27 | 1.7 | +H | 184.0735,86.0966,264.2668,344.1971 |
| 6 | 5.78 | Fuziline | C_24_H_39_NO_7_ | 453.273 | 454.281 | 1.7 | +H | 114.0928,96.0828,228.1579,322.2477 |
| 7 | 6.08 | Karakoline | C_22_H_35_NO_4_ | 377.257 | 378.264 | 1.2 | +H | 360.2549,134.8924,152.9054,224.8326 |
| 8 | 6.17 | Cammaconine | C_23_H_37_NO_5_ | 407.267 | 408.275 | 1.7 | +H | 358.2410,390.2651,134.8924,376.2488,152.9054 |
| 9 | 6.64 | Aconine | C_25_H_41_NO_9_ | 499.278 | 500.286 | 1.6 | +H | 136.9310,118.9194,84.9610,160.0080,188.0038 |
| 10 | 6.73 | Dictysine | C_21_H_33_NO_3_ | 347.246 | 348.254 | 2.3 | +H | 134.8924,152.9054,58.0658,118.9194,72.9370,84.9610,170.9181 |
| 11 | 6.74 | Songorine | C_22_H_31_NO_3_ | 357.23 | 358.238 | 1.5 | +H | 342.2416,134.8924,152.9054,118.9232,136.9310,84.9610,97.9681 |
| 12 | 6.82 | Isoboldine | C_19_H_21_NO_4_ | 327.147 | 328.155 | 2 | +H | 134.8924,152.9054,118.9232,105.0712,136.9310,98.0970,58.0658,72.9370,214.1580 |
| 13 | 6.86 | 12-epinapelline | C_22_H_33_NO_3_ | 359.246 | 360.254 | 1.3 | +H | 342.2416,134.8924,152.9054,118.9232,324.2340,98.0970,105.0712,232.8747 |
| 14 | 6.94 | Hypaconine | C_24_H_39_NO_8_ | 469.268 | 470.276 | 2.4 | +H | 360.2549,342.2416 |
| 15 | 7.57 | Deltamine | C_25_H_39_NO_7_ | 465.273 | 466.28 | 0 | +H | 134.8924,152.9054,118.9232,170.9135,84.9610,406.2592,206.8230,360.2549 |
| 16 | 7.93 | Lycoctonine | C_25_H_41_NO_7_ | 467.288 | 468.295 | -0.7 | +H | 454.2842,134.8924,152.9054,118.9232,436.2737,404.2406,170.9135,378.2648 |
| 17 | 8.14 | Flavadine | C_24_H_35_NO_5_ | 417.252 | 418.259 | 0.9 | +H | 134.8924,152.9054,400.2537,118.9194,408.2757,84.9610,170.9135,376.2488,206.8281,340.2268 |
| 18 | 8.63 | Foresticine | C_24_H_39_NO_6_ | 437.278 | 438.285 | 0.9 | +H | 96.9850,118.9232,136.9310,84.9610,188.0375,232.0272,362.2477 |
| 19 | 8.74 | Karakomine | C_22_H_33_NO_3_ | 359.246 | 360.253 | -0.9 | +H | 118.9232,136.9351,84.9610,134.8924,152.9054,342.2416 |
| 20 | 9.08 | Delsoline | C_25_H_41_NO_7_ | 467.288 | 468.296 | 1.5 | +H | 184.0735,104.1069,86.0966,124.9998,264.2668,258.1144,362.2611,342.3132,240.1031,272.6601,340.6228,222.9952 |
| 22 | 9.26 | Pseudaconine | C_25_H_41_NO_8_ | 483.283 | 484.291 | 0.7 | +H | 452.2617,438.2822,134.8924,152.9054,118.9194,140.9537,170.9181,232.8747,72.0802,250.8797 |
| 23 | 9.34 | Elasine | C_26_H_39_NO_8_ | 493.268 | 494.276 | 2.1 | +H | 356.2639,374.2791,105.0712 |
| 24 | 9.9 | Sachaconitine | C_23_H_37_NO_4_ | 391.272 | 392.28 | 1.3 | +H | 360.2549,134.8924,152.9054,328.2248,105.0712,310.2166,188.9070,240.8779,314.2390,252.8283 |
| 25 | 10.06 | Atisine | C_22_H_33_NO_2_ | 343.251 | 344.259 | 1.7 | +H | 134.8924,152.9054,170.9135, 72.0832, 122.9431, 206.8230,240.8833,252.8283 |
| 26 | 10.18 | Talatisamine | C_24_H_39_NO_5_ | 421.283 | 422.291 | 1.5 | +H | 136.9310,118.9232,84.9610,152.9881,174.8782,74.0985,156.8945,192.8882,390.2651,214.9878,360.2539 |
| 27 | 10.35 | Condelphine | C_25_H_39_NO_6_ | 449.278 | 450.286 | 1.6 | +H | 422.2924,390.2651,134.8924,152.9054,118.9232 |
| 28 | 10.99 | Eldelidine | C_25_H_39_NO_7_ | 465.273 | 466.281 | 1.2 | +H | 136.9310,118.9232,84.9610,174.8782,152.9054,422.2924,192.8833,254.8463,214.9207 |
| 29 | 11.03 | Hispaconitine | C_26_H_41_NO_8_ | 495.283 | 496.291 | -0.1 | +H | 184.0735,378.2921 |
| 30 | 11.09 | Vilmoraconitine | C_23_H_33_NO_3_ | 371.246 | 372.253 | -0.7 | +H | 300.2345,134.8924,152.9054,118.9194,84.9610,170.9135 |
| 31 | 11.37 | Lucidusculine | C_24_H_35_NO_4_ | 401.257 | 402.265 | 2 | +H | 184.0735,378.2853,112.0278 |
| 32 | 11.42 | Deltaline | C_27_H_41_NO_8_ | 507.283 | 508.291 | 1.2 | +H | 480.3006,462.2906,134.8924,152.9054,430.2611 |
| 33 | 11.52 | Delcorine | C_26_H_41_NO_7_ | 479.288 | 480.296 | 1.2 | +H | 462.2831,430.2611,154.1217,398.2360,108.0822,58.0658,404.2476,122.0980,134.8924 |
| 34 | 11.7 | Chasmanine | C_25_H_41_NO_6_ | 451.293 | 452.301 | 1.1 | +H | 136.9310,118.9232,84.9610,436.3335,322.2540 |
| 35 | 11.89 | Cheilanthifoline | C_19_H_19_NO_4_ | 325.131 | 326.139 | 0.9 | +H | 134.8924,152.9054,118.9232,170.9181,84.9610, 280.0941,232.8693 |
| 36 | 12 | 14-O-acetylneoline | C_26_H_41_NO_7_ | 479.288 | 480.296 | 0.8 | +H | 136.9310,118.9232,84.9610,454.3473,152.9054,172.9341,322.2477,436.3335,462.2906,356.2406,72.9911,220.8881 |
| 37 | 12.87 | Delphatine | C_26_H_43_NO_7_ | 481.304 | 482.312 | 0.6 | +H | 86.0966,173.1285,136.9310,308.2431,336.2417,354.2507,432.1738,476.2763 |
| 38 | 15 | Delbrusine | C_27_H_43_NO_7_ | 493.304 | 494.312 | 1.5 | +H | 462.2831,134.8924,152.9054,430.2611,84.9610, 398.2290,108.0822,188.9070,404.2476,206.8230,232.8693,240.8779,282.0548,360.2406 |
| 39 | 16.7 | Benzoylmesaconine | C_31_H_43_NO_10_ | 589.289 | 590.296 | 0.3 | +H | 540.2565,105.0351,558.2729,508.2304,572.2843,526.2417 |
| 41 | 17.63 | Lappaconitine | C_32_H_44_N_2_O_8_ | 584.31 | 585.318 | 1 | +H | 574.2977,136.9310,152.9054,542.2783,202.9102,528.3019 |
| 42 | 17.68 | 8-O-Ethylyunaconitine | C_31_H_43_NO_9_ | 573.294 | 574.301 | 0.2 | +H | 542.2783,118.9232,136.9310,152.9054,105.0351,84.9610,206.8230,270.8157,524.2661,322.8164,468.9792 |
| 44 | 19.31 | Benzoylaconine | C_32_H_45_NO_10_ | 603.304 | 604.312 | 1 | +H | 105.0351,554.2732,572.2843,522.2460,586.2989,540.2565 |
| 46 | 20.5 | Acsonine | C_31_H_41_NO_8_ | 555.283 | 556.29 | -0.1 | +H | 538.2792,105.0351,506.2573,524.2661,136.9310,152.9054,474.2284,492.2408 |
| 47 | 21.23 | 16-epi-pyroaconitine | C_32_H_43_NO_9_ | 585.294 | 586.301 | 0.5 | +H | 114.0928,84.9610,174.8782,360.2473,576.4216,326.1755,454.3398,498.0453,522.6722 |
| 48 | 22.16 | Pseudaconitine | C_36_H_51_NO_12_ | 689.341 | 690.349 | 1.3 | +H | 586.2989,136.9310,118.9232,618.3312,336.2545,554.2815 |
| 49 | 22.46 | Mesaconitine | C_33_H_45_NO_11_ | 631.299 | 632.307 | 0.4 | +H | 572.2843,354.1705,512.2678,540.2565 |
| 50 | 22.72 | Hetisine | C_20_H_27_NO_3_ | 329.199 | 330.206 | -0.8 | +H | 136.9310,118.9194,105.0351,152.9054,84.9610, 280.0941,178.8977,208.8621,240.8833,312.2028,258.8668,292.8550,302.14367,318.1271 |
| 51 | 22.97 | 14-Benzoylneoline | C_31_H_43_NO_7_ | 541.304 | 542.311 | 0 | +H | 136.9310,118.9194,152.9054,105.0351,510.2868,84.9610,170.9135,278.0941,492.2798,360.2406,220.9195,240.8779 |
| 52 | 24.24 | Aconifine | C_34_H_47_NO_12_ | 661.31 | 662.318 | 1.5 | +H | 586.2989,136.9310,118.9194,536.2649,602.2949,105.0351 |
| 53 | 24.51 | Szechenyine | C_36_H_51_NO_11_ | 673.346 | 674.355 | 1.8 | +H | 572.3264,136.9310,586.2989,118.9232,366.1960 |
| 54 | 24.63 | Hypaconitine | C_33_H_45_NO_10_ | 615.304 | 616.313 | 1.4 | +H | 556.2879,524.2661,338.1727 |
| 55 | 25.16 | Jesaconitine | C_35_H_49_NO_12_ | 675.325 | 676.334 | 2.3 | +H | 586.2989,570.3080,136.9310,118.9232,105.0351,536.2649,410.2330,279.0941,524.2661,644.3115 |
| 56 | 25.6 | Flavaconidine | C_32_H_41_NO_12_ | 631.263 | 632.272 | 2.7 | +H | 602.3295,135.0437,136.9310,118.9232,552.2953,570.3080 |
| 57 | 26.51 | Falconeridine | C_34_H_49_NO_9_ | 615.341 | 616.348 | -0.8 | +H | 136.9310,118.9194,140.9537,152.9054,602.2949,584.3241,220.9195 |
| 58 | 27.46 | Aconitine | C_34_H_47_NO_11_ | 645.315 | 646.323 | 1 | +H | 616.3119,586.2989,556.2879,368.1848 |
| 59 | 27.87 | 3-Deoxyjesaconitine | C_35_H_49_NO_11_ | 659.331 | 660.338 | 0.7 | +H | 602.3295,570.3080,105.0351 |
| 60 | 27.9 | Neojiangyouaconitine | C_33_H_47_NO_9_ | 601.325 | 602.333 | 0.4 | +H | 105.0351,550.2796,538.2792,506.2573,474.2284,518.2576,384.2208 |
| 61 | 28.67 | Acoforesticine | C_33_H_47_NO_8_ | 585.33 | 586.338 | 0.6 | +H | 554.3146,118.9194 |
| 64 | 29.16 | 3-Acetylaconitine | C_36_H_49_NO_12_ | 687.325 | 688.333 | 0.8 | +H | 136.9310,118.9232,628.3187,524.3064,646.3225,552.3283,568.2933 |
| 67 | 29.46 | Chasmaconitine | C_34_H_47_NO_9_ | 613.325 | 614.333 | 0.6 | +H | 554.3146,522.2863,586.3415,490.2600,136.9310,105.0351,152.9054,458.2372,368.2253,400.2537,224.9065,254.8463 |
| 68 | 29.91 | 3-Deoxyaconitine | C_34_H_47_NO_10_ | 629.32 | 630.329 | 2 | +H | 570.3080,538.2792,352.1935 |
| 69 | 30.12 | 3-O-Anisoyljesaconitine | C_43_H_55_NO_14_ | 809.362 | 810.371 | 2 | +H | 630.3311,136.9310,570.3080,118.9232,644.3472,538.2792,352.1935,492.2798 |
| 71 | 30.61 | Penduline | C_34_H_47_NO_9_ | 613.325 | 614.333 | 0.4 | +H | 86.0966,70.0662,132.1038,582.6311,120.0809,582.3016,268.1301,476.2686 |
| 73 | 33.12 | Foresaconitine | C_35_H_49_NO_9_ | 627.341 | 628.348 | 0.2 | +H | 568.3268,536.2975,136.9310,118.9194,140.9537,504.2801 |
| 85 | 37.81 | Aldohypaconitine | C_33_H_43_NO_11_ | 629.284 | 630.293 | 2.6 | +H | 136.9310,118.9232,360.2406,272.8518,510.2550,570.2744,478.2286 |
| 97 | 49.05 | 8-O-Linoleoyl-14-anisoylaconine | C_51_H_77_NO_12_ | 895.545 | 896.55 | -2.1 | +H | 184.0735,136.9310,105.0712,298.3476,628.5341,736.9242,470.3298,558.3173,786.6075 |
| 109 | 56.29 | 12-acetyllucidusculine | C_26_H_37_NO_5_ | 443.267 | 444.276 | 2.5 | +H | 184.0735,398.3110,368.4212 |
| 113 | 58.54 | Guan-fu base A | C_24_H_31_NO_6_ | 429.215 | 430.221 | -3.9 | +H | 184.0735,270.2775,158.1530,86.0966,264.2668 |
| 126 | 69.79 | Nominine | C_28_H_39_NO | 405.303 | 406.309 | -2.6 | +H | 114.0928,136.9310,84.9610 |
| 82 | 36.25 | Oleanolic acid | C_30_H_48_O_3_ | 456.3604 | 457.3683 | 1.5 | +H | 201.0451 |
| 95 | 47.88 | Maslinic acid | C_30_H_48_O_4_ | 472.3553 | 473.3626 | 0.2 | +H | 455.3235 |
| 106 | 51.86 | Betulin | C_30_H_50_O_2_ | 442.3811 | 443.3892 | 1.9 | +H | 407.3662 |
| 62 | 28.83 | α-Santalol | C_15_H_24_O | 220.183 | 221.191 | 2.6 | +H | 201.0501,95.0853,77.0407,81.0696,69.0714 |
| 63 | 28.89 | 3-Epizaluzanin C | C_15_H_18_O_3_ | 246.126 | 247.134 | 2.4 | +H | 229.0451 |
| 66 | 29.39 | Reynosin-1 | C_15_H_20_O_3_ | 248.141 | 249.149 | 1.3 | +H | 89.0598,231.0883 |
| 70 | 30.54 | Costunolide | C_15_H_20_O_2_ | 232.146 | 233.154 | 2.9 | +H | 187.1508 |
| 74 | 33.26 | 11β, 13-dihydroxylinolide | C_15_H_22_O_2_ | 234.162 | 235.17 | 1.3 | +H | 217.1599 |
| 75 | 34.79 | Acorusdiol | C_15_H_24_O | 220.183 | 221.19 | 1.4 | +H | 136.9310,118.9194,69.0685,201.0451,149.0235 |
| 78 | 35.48 | Calamusenone | C_15_H_22_O | 218.167 | 219.175 | 3.2 | +H | 184.0735,86.0966,124.9998,104.1069,146.9818,163.0172 |
| 79 | 35.48 | 2-hydroxyacorenone | C_15_H_24_O_2_ | 236.178 | 237.185 | 1.3 | +H | 136.9310,118.9194,181.0879,201.0451,91.0531,219.1034 |
| 81 | 36.03 | (-)-Cadala-1,4,9-triene | C_15_H_22_ | 202.172 | 203.18 | 2.4 | +H | 136.9310,118.9232,77.0376,84.9610,105.0712,149.0235,91.0531,152.9054 |
| 84 | 36.81 | Lactin diol | C_15_H_24_O_2_ | 236.178 | 237.185 | 2.1 | +H | 165.1263,136.9310,91.0565,95.0853,118.9232,105.0712,123.0836,140.9537,201.0451,81.0696,107.0860,149.0235 |
| 87 | 42.59 | Alantolactone | C_15_H_20_O_2_ | 232.146 | 233.154 | 1.7 | +H | 205.0712,187.1460,215.1426 |
| 93 | 47.45 | Bullatantriol | C_15_H_28_O_3_ | 256.204 | 257.212 | 3.8 | +H | 184.0735,136.9310,118.9232,159.1191,201.0501,77.0407,95.0853,101.9499,146.0831,163.0397 |
| 94 | 47.81 | Bergamotol | C_15_H_24_O | 220.183 | 221.19 | 0.8 | +H | 184.0735,136.9310,118.9232,81.0696,105.0712,91.0565,201.0501,77.0407,149.0235,69.0714 |
| 96 | 48.17 | Costic acid | C_15_H_22_O_2_ | 234.162 | 235.17 | 1.8 | +H | 184.0735,104.1069,86.0966,124.9998 |
| 98 | 49.06 | (-)-Cyperene | C_15_H_24_ | 204.188 | 205.196 | 2.1 | +H | 184.0735,136.9310,81.0696,95.0853,105.0712,118.9232,131.0867,145.1025,67.0541,107.0860,77.0407 |
| 99 | 49.35 | Calamenene | C_15_H_22_ | 202.172 | 203.18 | 2.8 | +H | 184.0735,136.9310,118.9232,105.0712,81.0696,77.0407,67.0541,149.0235 |
| 100 | 49.85 | α-荜澄茄油烯 | C_15_H_24_ | 204.188 | 205.194 | -3.8 | +H | 184.0735,105.0712,119.0844,136.9310,147.1183,91.0565,81.0696,133.1004,69.0714,77.0407,67.0541 |
| 103 | 51.57 | α-榄香烯 | C_15_H_24_ | 204.188 | 205.196 | 1.9 | +H | 184.0735,136.9310,95.0853,107.0860,118.9194,81.0696,105.0712,177.0578,77.0407,133.1004,140.9537,149.0235,69.0685 |
| 104 | 51.69 | Espatulenol | C_15_H_24_O | 220.183 | 221.19 | -2.1 | +H | 184.0735,95.0853,109.1015,121.1009,81.0696,119.0844,135.1173,133.1004,91.0565,159.1191 |
| 105 | 51.77 | α-香附酮 | C_15_H_22_O | 218.167 | 219.175 | 2.3 | +H | 184.0735,95.0853,107.0860,121.1009,81.0696,145.1025,159.1191,133.1004,77.0407,69.0685,111.0802,129.0683,149.1352,161.1315 |
| 107 | 53.43 | Costol | C_15_H_24_O | 220.183 | 221.19 | 1.9 | +H | 184.0735,89.0598,133.0883,109.1015,81.0696,177.1140,119.0844,147.1183,69.0714,135.1173,59.0322 |
| 108 | 54.12 | (-)-Caryophyllene oxide | C_15_H_24_O | 220.183 | 221.19 | 1.2 | +H | 184.0735,97.0647,89.0598,109.0647,133.0883,81.0696,69.0714 |
| 122 | 65.61 | 1-hydroxyepiacorone | C_15_H_24_O_3_ | 252.173 | 253.18 | -0.1 | +H | 118.9232,136.9310,84.9610 |
| 123 | 66.98 | Acorusdiol | C_15_H_24_O_3_ | 252.173 | 253.181 | 5 | +H | 184.0735,59.0322,73.0483,95.0853,109.1015,121.1009,133.0883 |
| 128 | 69.81 | β-sesquiphellandrene | C_15_H_24_ | 204.188 | 205.196 | 2.1 | +H | 69.0714,83.0873,97.1029,184.0735,135.1173,109.1015,121.1009 |
| 1 | 0.79 | Methyl palmitate | C17H34O2 | 270.256 | 271.264 | 1.7 | +H | 184.0735,86.0966,124.9998,146.9818,95.0887,97.1029,104.1069,69.0714,146.0874,81.0727,71.0860,57.0700,79.0548,89.0632,93.0718,109.1052,114.0928,121.1009,133.0883,149.1352,162.9543,166.0635,175.1249,60.0830,67.0541 |
| 21 | 9.14 | Safrole | C10H10O2 | 162.068 | 163.076 | 4 | +H | 134.8924,152.9054,118.9232,108.0822,140.9537,84.9610,58.0658,77.0407,149.0235,113.9651,97.9681,72.0802,155.0841 |
| 40 | 17.27 | (E)-methyl isoeugenol | C11H14O2 | 178.099 | 179.107 | 2.6 | +H | 134.8924,152.9054,118.9194,140.9537,84.9610,149.0235,122.9431,72.9370,77.0407,148.9118,113.9613,91.0531,97.9716,101.9499,158.9638,163.0441,102.9434,96.9608,71.9309 |
| 43 | 19.04 | Acoramone | C12H16O4 | 224.1049 | 225.1127 | 2.3 | +H | 114.0928,136.9310,96.0828,209.1673,174.8828 |
| 45 | 20.25 | beta-asarone | C12H16O3 | 208.1099 | 209.1176 | 1.6 | +H | 136.9310,152.9054,179.0719,91.0565,194.0921, 181.0879,123.0446 |
| 65 | 29.17 | Androstere-4-ene-17-keto-3β-acetate | C21H30O3 | 330.22 | 331.225 | -4.1 | +H | 136.9310,118.9232,201.0451,152.9054,84.9610,279.0941,220.8777 |
| 72 | 31.51 | α-ionone | C13H20O | 192.151 | 193.159 | 1.7 | +H | 136.9310,118.9232,140.9537,105.0712,77.0407 |
| 76 | 35.09 | Elemicin | C12H16O3 | 208.1099 | 209.1174 | 0.8 | +H | 179.0719,151.0750,91.0565,194.0921,121.0661 |
| 77 | 35.33 | Isodehydroandrosterone 3-acetate | C21H30O3 | 330.22 | 331.227 | -0.2 | +H | 136.9310,118.9194,201.0501,91.0531,279.0941,311.1407 |
| 80 | 35.58 | Methyl eugenol | C11H14O2 | 178.099 | 179.107 | 1.7 | +H | 136.9310,118.9194,140.9537,84.9610,91.0531,151.0750,77.0376,107.0496,101.9499,122.9431,116.9783,131.0504,74.0985,164.0836,72.0832 |
| 88 | 43.53 | Bornyl acetate | C12H20O2 | 196.146 | 197.154 | 3.2 | +H | 109.0647,97.0647,136.9310,143.0837,128.0630,129.0683,91.0531,118.9232,157.1017,119.0883,81.0696,115.0569,155.0885,77.0407 |
| 89 | 43.74 | Ermanthin | C15H18O2 | 230.131 | 231.138 | 1.7 | +H | 184.0735,136.9310,118.9232,104.1069,84.9610,192.9615 |
| 92 | 46.3 | β-ionone | C13H20O | 192.151 | 193.159 | 1.5 | +H | 136.9310,118.9232,140.9537,84.9610,91.0531,105.0712,77.0407,119.0844,149.0235,107.0860,122.9431,134.8924,67.0541,165.0721 |
| 101 | 50.38 | Guaiazulene | C15H18 | 198.141 | 199.149 | 1.8 | +H | 105.0712,91.0565,81.0696,119.0844,136.9310,77.0407,143.0879,157.1017,67.0541,86.0966,165.0721 |
| 102 | 51.52 | Normuscone | C15H28O | 224.214 | 225.221 | 0.3 | +H | 184.0735,136.9310,97.0647,118.9232,119.0844,77.0376,81.0696,149.0235,159.1191,201.0501,121.1009,67.0541,185.0773,173.1331,177.0578,201.1649 |
| 110 | 57.28 | Androstenediol | C19H30O2 | 290.225 | 291.232 | 1.8 | +H | 184.0735,295.1958,131.0021,231.1437,112.0278,95.0853,71.0860 |
| 111 | 58.02 | (-)-sabinene | C10H16 | 136.125 | 137.133 | 2.6 | +H | 69.0714,83.0873,97.1029,55.0548,93.0718,107.0860,111.1173,121.1009,100.0757,57.0700,67.0541,89.0598 |
| 112 | 58.28 | palmitic acid | C16H32O2 | 256.24 | 257.248 | 3.2 | +H | 184.0735,89.0598,133.0883,95.0853,71.0860,81.0696,177.1140,57.0700,109.1015 |
| 114 | 59.24 | Isocembrol | C20H34O | 290.261 | 291.269 | 2.8 | +H | 136.9310,118.9194,84.9610,247.1432,182.0978,255.1160 |
| 115 | 60.86 | Muscone | C16H30O | 238.23 | 239.237 | 1.6 | +H | 184.0735,131.0021,231.1437,112.0278,95.0853,223.0478,71.0860,81.0696,85.1005,57.0700,109.1015,231.6468,184.1403 |
| 116 | 62.09 | Myristic acid | C14H28O2 | 228.2089 | 229.2168 | 2.5 | +H | 184.0735,73.0483,86.0966,89.0598,59.0322,133.0883 |
| 117 | 62.68 | Elaidic acid | C18H34O2 | 282.256 | 283.264 | 3.5 | +H | 184.0735,59.0322,73.0483,86.0966,95.0853,133.0883 |
| 120 | 64.11 | 5α -androstere-3 β,17α -diol | C19H32O2 | 292.24 | 293.247 | -0.3 | +H | 184.0735,104.1069,86.0966 |
| 124 | 68.61 | (3E,12Z)-1,3,12-Nonadecatriene-5,14-diol | C19H34O2 | 294.256 | 295.264 | 1.4 | +H | 184.0735,86.0966,124.9998,104.1069,146.9818,162.9498,81.0696,95.0853 |
| 125 | 69.6 | α-asarone | C12H16O3 | 208.1099 | 209.1178 | 2.7 | +H | 184.0735,69.0714,83.0873,97.1029,121.1009,55.0548,135.1173,89.0598,149.1352 |
| 127 | 69.81 | Aplotaxene | C17H28 | 232.219 | 233.227 | 2.2 | +H | 69.0714,83.0873,97.1029,184.0735,135.1173,109.1015,121.1009,100.0757,55.0548 |
| 83 | 36.77 | Cubebin | C20H20O6 | 356.126 | 357.1321 | -3.3 | +H | 165.1263,136.9310,105.0712,91.0565,118.9194,79.0548,140.9537,275.1406,67.0541,208.1103,65.0383,194.0921,217.0563,259.1669,338.1274,237.1898,321.1434 |
| 86 | 42.58 | (Z)-Ligustilide | C12H14O2 | 190.0994 | 191.107 | 1.9 | +H | 173.0961 |
| 90 | 44.43 | Tianshic acid | C18H34O5 | 330.2406 | 331.2481 | 0.6 | +H | 136.9310,97.0647,118.9232,120.0809,184.0735,140.9537,84.9610,201.0501,77.0376,120.9538,149.0235,72.0832,163.0397,178.9024,279.0941,220.9195,247.1487,199.1489,281.0607,301.1436 |
| 91 | 45.1 | Veraguensin | C22H28O5 | 372.1937 | 373.2017 | 2 | +H | 136.9310,296.2971,118.9232,140.9537,184.0735,201.0501,91.0565,105.0712,119.0883,77.0407,145.1025,159.1191,279.0941,330.3380,165.0901,297.2972,67.0541,286.3111,220.9195,232.8586,254.8520,272.8518 |
| 118 | 63.03 | Cycloartenol | C30H50O | 426.3862 | 427.3935 | 0.1 | +H | 184.0735,284.2964,69.0714,95.0853,109.1015,285.2999,135.1173,156.8901,185.0773,409.3854,264.2668 |
| 119 | 63.76 | Stearic acid | C18H36O2 | 284.2715 | 285.28 | 4 | +H | 184.0735,71.0860,95.0853,109.1015,131.0021,185.0773,57.0700,81.0696,264.2725 |
| 121 | 65.35 | PENTADECANOIC ACID | C15H30O2 | 242.2246 | 243.2317 | -0.5 | +H | 184.0735,59.0322,86.0966,89.0598,73.0483,133.0883 |

**Tab.S5. Component identification results of WPW under UPLC-Q-TOF-MS positive/negative ion mode**

| **Peak no.** | **T_R_/min** | **Identity** | **Molecular formula** | **Theoretical (Da)** | **Calculated mass (Da)** | **Error (ppm)** | **Adducts** | **MS Fragmentation** |
| --- | --- | --- | --- | --- | --- | --- | --- | --- |
| 1 | 27.37 | Chebuloside I | C_36_H_58_O_10_ | 650.403 | 695.4045 | 4.7 | +HCOO | 487.3456,160.8413,78.9598,174.9563,180.9180,214.8733 |
| 2 | 30.71 | Ajiang elemin | C_30_H_48_O_6_ | 504.3451 | 503.3403 | 4.9 | -H | 485.3355,409.3123,473.2243 |
| 3 | 40.05 | Arjunolic acid | C_30_H_48_O_5_ | 488.3502 | 487.3449 | 4.1 | -H | 160.8413,174.9563,78.9598,146.9656,130.9668,61.9883,180.9132,96.9607,425.3438,191.9495,254.8613 |
| 4 | 54.06 | Corosolic acid | C_30_H_48_O_4_ | 472.3553 | 471.3484 | 0.9 | -H | 427.3566,146.9656,116.9264,96.9607,174.9563,137.0983,411.3297,61.9883,207.1391,357.3148,69.0357,99.0441,160.8413 |
| 5 | 56.22 | Ursolic acid | C_30_H_48_O_3_ | 456.3604 | 455.3541 | 2.2 | -H | 175.0401,193.0481,146.9656,325.1824,134.0374,116.9302,61.9883,160.0167,96.9607,99.9251,277.2200,208.9360,78.9598 |
| 6 | 56.3 | Aplotaxene | C_17_H_28_ | 232.2191 | 277.218 | 2.6 | +HCOO | 146.9656,174.9563,183.0115,116.9302,251.1667 |
| 7 | 56.97 | Betulinic acid | C_30_H_48_O_3_ | 456.3604 | 455.355 | 4.3 | -H | 193.0481,175.0401,134.0374,146.9656,96.9607,116.9302,61.9883,183.0115,325.1888 |
| 8 | 58.61 | 2,2'-Methylenebis | C_23_H_32_O_2_ | 340.2402 | 339.233 | 0.1 | -H | 163.1121 |
| 9 | 58.8 | Methyl betulinate | C_31_H_50_O_3_ | 470.376 | 515.3763 | 4.2 | +HCOO | 279.2335,146.9656,116.9302,409.1912,174.9563,280.2341,175.0401,399.1589,426.1788,325.1824 |
| 10 | 58.9 | Linoleic acid | C_18_H_32_O_2_ | 280.2402 | 279.233 | 0.3 | -H | 261.2322 |
| 11 | 61.3 | Pentadecene | C_15_H_30_ | 210.2348 | 255.2334 | 1.9 | +HCOO | 116.9264,146.9656,61.9883,174.9563,96.9607,134.8944,130.9668,102.9342,183.0115,191.9495,200.8811 |
| 12 | 68.72 | Daucosterol | C_35_H_60_O_6_ | 576.439 | 621.4385 | 2.1 | +HCOO | 146.9656,116.9264,99.9286,174.9563,191.9447,349.1727,255.2322 |
